# Narrow-Line-Width Electroluminescence in Monolayer WSe_2_ Enabled by a Sub‑5 nm Au/Pt Nanogap

**DOI:** 10.1021/acs.nanolett.6c03133

**Published:** 2026-10-03

**Authors:** Shurong Miao, I. K. M. Reaz Rahman, Aditya Nirmale, Inha Kim, Dorottya Urmossy, Thomas P. Darlington, Harishankar Jayakumar, Seiichiro Izawa, Archana Raja, Daryl C. Chrzan, Yutaka Majima, Ali Javey

**Affiliations:** † Electrical Engineering and Computer Sciences, 1438 University of California, Berkeley, Berkeley, California 94720, United States; ‡ Materials and Structures Laboratory, Institute of Science Tokyo, Yokohama 2268503, Japan; § Materials Sciences Division, Lawrence Berkeley National Laboratory, Berkeley, California 94720, United States; ∥ The Molecular Foundry, Lawrence Berkeley National Laboratory, Berkeley, California 94720, United States; ⊥ Department of Materials Science and Engineering, University of California, Berkeley, Berkeley, California 94720, United States; # Kavli Energy NanoScience Institute at the University of California, Berkeley, Berkeley, California 94720, United States

**Keywords:** AC electroluminescence, WSe_2_ monolayer, narrow-line-width emission, nanogap electrodes, localized emitters, two-dimensional nanophotonics

## Abstract

Spectrally isolated, electrically driven emission from localized states in two-dimensional semiconductors remains challenging in scalable planar devices. Here, we report narrow-line-width alternating-current electroluminescence (ACEL) from localized states in monolayer WSe_2_ using a lithographically defined sub-5 nm Au/Pt nanogap on SiO_2_/Si at 3.5 K. The curved nanogap geometry produces a nonuniform strain landscape and regions of enhanced electric field that may influence the local electronic environment. Under square-wave gate excitation, the device exhibits a spectrally isolated ACEL line at 1.652 eV with an apparent fitted line width of approximately 2 meV, in contrast to broad, multipeak photoluminescence from the same device. Spectral decomposition identifies dominant ZPL-like and weaker red-shifted sideband-like contributions. This nanogap architecture provides a scalable planar platform for narrow-line-width, electrically driven localized emission in two-dimensional materials.

## Graphical abstract

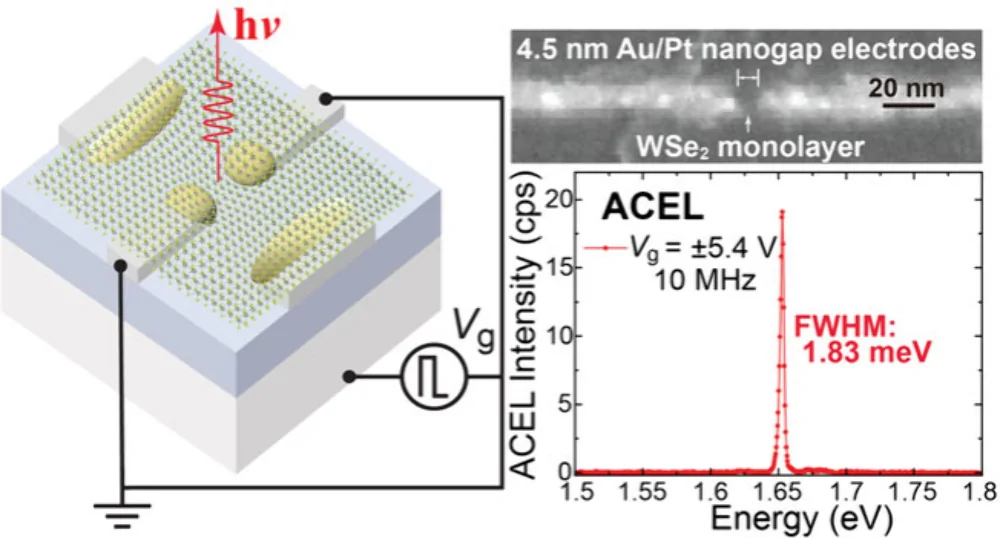

Electrically driven narrow-line-width light emitters are crucial components for scalable on-chip quantum photonic technologies, including quantum communication, computation, and metrology.^1–5^ Solid-state single-photon sources based on semiconductor quantum dots and color centers have made significant advances,^4–7^ yet integrating them into planar architectures with direct electrical addressability remains challenging,^8^ motivating the search for alternative material platforms. Monolayer tungsten diselenide (WSe_2_) has emerged as a promising candidate in this area due to its direct bandgap, strong excitonic effects, and atomically thin geometry, which makes it compatible with planar device integration. It hosts localized excitonic states at native strained point defects^9–11^ that give rise to discrete narrow-line-width emission at cryogenic temperatures. Strain engineering has become the primary route to positioning such emission centers at defined locations, typically by conforming the monolayer over dielectric or metallic nanopillars^12–19^ or coupling it to photonic cavities,^20–24^ where local lattice deformation creates potential funnels that trap excitons. Plasmonic nanostructures have further been explored to combine strain-induced localization with near-field emission enhancement.^25–27^

Despite this progress, optically pumped approaches carry fundamental limitations for on-chip integration. A diffraction-limited laser spot (∼1 μm) inevitably illuminates many localized states simultaneously, producing congested multipeak spectra that preclude addressing a specific emission center, and an external laser source is inherently incompatible with integrated circuits. Electrical pumping offers a direct solution. The predominant approach has been direct-current (DC) vertical tunneling in van der Waals heterostructures, where WSe_2_ is encapsulated between hexagonal boron nitride (hBN) spacers and contacted by graphene electrodes.^28–30^ While sub-meV line widths have been achieved in such heterostructure devices,^30^ the multistep mechanical transfer and precise layer alignment required limit device yield and scalability. Critically, DC vertical tunneling without lateral geometric confinement allows carrier flow across the broad planar interface, simultaneously activating multiple localized states. Square-wave alternating-current (AC) excitation of monolayer transition-metal dichalcogenides (TMDs) offers a geometrically simpler alternative, where abrupt voltage transients drive sequential electron and hole injection that recombine radiatively in the semiconductor channel.^31–33^ However, existing AC devices operate in large-area geometries and produce broad ensemble emission spanning tens of meV, as the absence of spatial confinement prevents selective addressing of individual defect states.

Here, we demonstrate a fully on-chip nanogap platform that generates narrow-line-width AC electroluminescence (ACEL) from monolayer WSe_2_ with an apparent fitted line width of approximately 2 meV, without requiring van der Waals layer stacking. Our planar architecture defines a sub-5 nm metallic junction using heteroepitaxial spherical Au/Pt (HS-Au/Pt) nanogap electrodes, formed by electron-beam lithography and electroless gold plating (ELGP),^34–37^ requiring only one monolayer transfer. The curved nanogap topography produces a highly nonuniform nanoscale strain landscape that may modify the local electronic environment, while the square-wave AC bottom gate voltage drives phase-dependent carrier injection. The spatial selectivity of this approach is evidenced by the contrast between the broad, multipeak photoluminescence (PL) and the narrow ACEL peak observed on the same device. Together, these features provide a scalable strategy for electrically addressing localized emission in 2D semiconductors.

We designed a solid-state nanogap architecture on a 50 nm SiO_2_/Si substrate (Figure 1a) based on electron-beam-lithographically defined Pt/Ti nanogaps. ELGP of the nanogap reduces the initial 12–15 nm Pt gap to below 5 nm.^34–37^ Subsequently, a monolayer of WSe_2_ was transferred onto the prepatterned nanogap electrodes using a dry-transfer technique.^34–37^ This configuration allows the monolayer to bridge the spherical nanogap electrodes, which is expected to produce localized strain near the junction. In this configuration, a 10 MHz square-wave AC voltage is applied to the global silicon back-gate to drive the emission, while both nanogap electrodes are grounded and act as a common reference.

**Figure 1:**
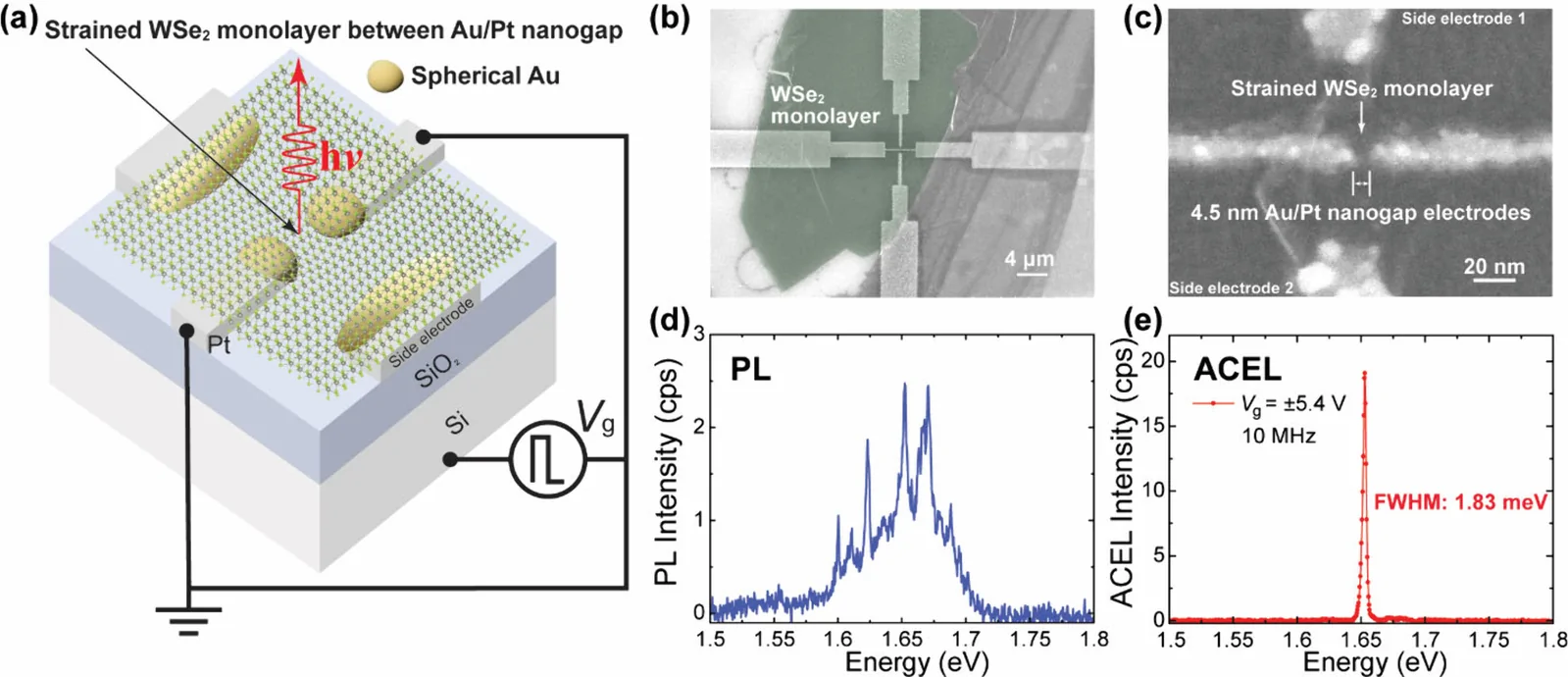
Device architecture and optical response of the sub-5 nm Au/Pt nanogap WSe_2_ emitter. (a) Schematic illustration of the device architecture. Four Pt/Ti nanowires form a cross-bar geometry, in which a pair of heteroepitaxially grown spherical Au/Pt nanoparticles defines a primary nanogap in the sub-5 nm regime. A monolayer of WSe_2_ is dry-transferred onto the electrodes, forming a geometry expected to generate a nonuniform strain landscape near the nanogap. A square-wave voltage is applied to the Si back gate, while the nanogap electrodes are grounded to drive ACEL. (b) Scanning electron microscopy (SEM) image of the device, showing the WSe_2_ monolayer (green overlay) on the four-terminal cross nanogap structure. (c) High-resolution SEM image of the central nanogap region, showing spherical Au/Pt electrodes with a gap of ∼4.5 nm and adjacent side electrodes separated by ∼35 nm, covered by a continuous WSe_2_ monolayer. (d) PL spectrum of the device under continuous-wave 532 nm laser excitation (35 nW, 3.5 K), exhibiting a broad, multipeak emission profile. (e) ACEL spectrum driven by a 10 MHz square-wave voltage (*V*_g_ = ±5.4 V, 3.5 K), showing a dominant spectrally isolated emission line with a line width of ∼2 meV.

Figure 1b shows a large-scale FE-SEM image confirming the structural integrity of the transferred WSe_2_ flake across the four-terminal cross architecture. The monolayer WSe_2_ region is highlighted in green. In the active nanogap region (Figure 1c), a 4.5 nm nanogap is observed alongside a much wider side-electrode (∼35 nm gap). The SEM image shows that the monolayer WSe_2_ spans the Au/Pt electrodes at the sub-5 nm junction, forming a geometry expected to produce a nonuniform strain distribution near the nanogap. Device fabrication details are provided in Methods and Figures S1 and S2. Prior to optical measurements, room-temperature direct-current transport confirmed electrical connectivity and showed typical *p*-type behavior (Figure S3).

The optical properties of the nanogap devices were characterized at 3.5 K. The PL spectrum acquired from the device under steady-state 532 nm laser excitation (∼35 nW) reveals a broad, complex emission band composed of multiple peaks spanning 1.60–1.70 eV (Figure 1d). Driven by a 10 MHz square-wave AC voltage (*V*_g_ = ±5.4 V), the ACEL spectrum (Figure 1e) exhibits a dominant narrow emission line centered at ∼1.652 eV with a full width at half-maximum (fwhm) of 1.83 meV. This behavior is reproducible, with sharp ACEL lines consistently observed alongside multipeak PL profiles (Figure S4). This contrast reflects the different excitation areas of PL and ACEL. The diffraction-limited laser spot (∼1 μm) samples spatial variations in the local electronic environment across the three-dimensional nanostructure and excites an ensemble of localized states, producing multipeak emission even at low excitation power. In contrast, the AC-driven nanogap concentrates carrier injection within the sub-5 nm junction and preferentially populates a smaller subset of localized states, producing the dominant narrow ACEL feature.

To characterize the voltage dependence of ACEL, we acquired emission spectra at 3.5 K under 10 MHz square-wave excitation while systematically varying the gate amplitudes *V*_g_. Figure 2a presents a 2D contour map of ACEL intensity as a function of emission energy and *V*_g_. The ACEL peak energy remains at 1.652 eV over the *V*_g_ range of ±4.6 V to ±5.8 V. As *V*_g_ increases, the ACEL intensity exhibits a pronounced threshold-like behavior, with a dominant, narrow emission line emerging at 1.652 eV once the driving voltage exceeds ±4.6 V.

**Figure 2:**
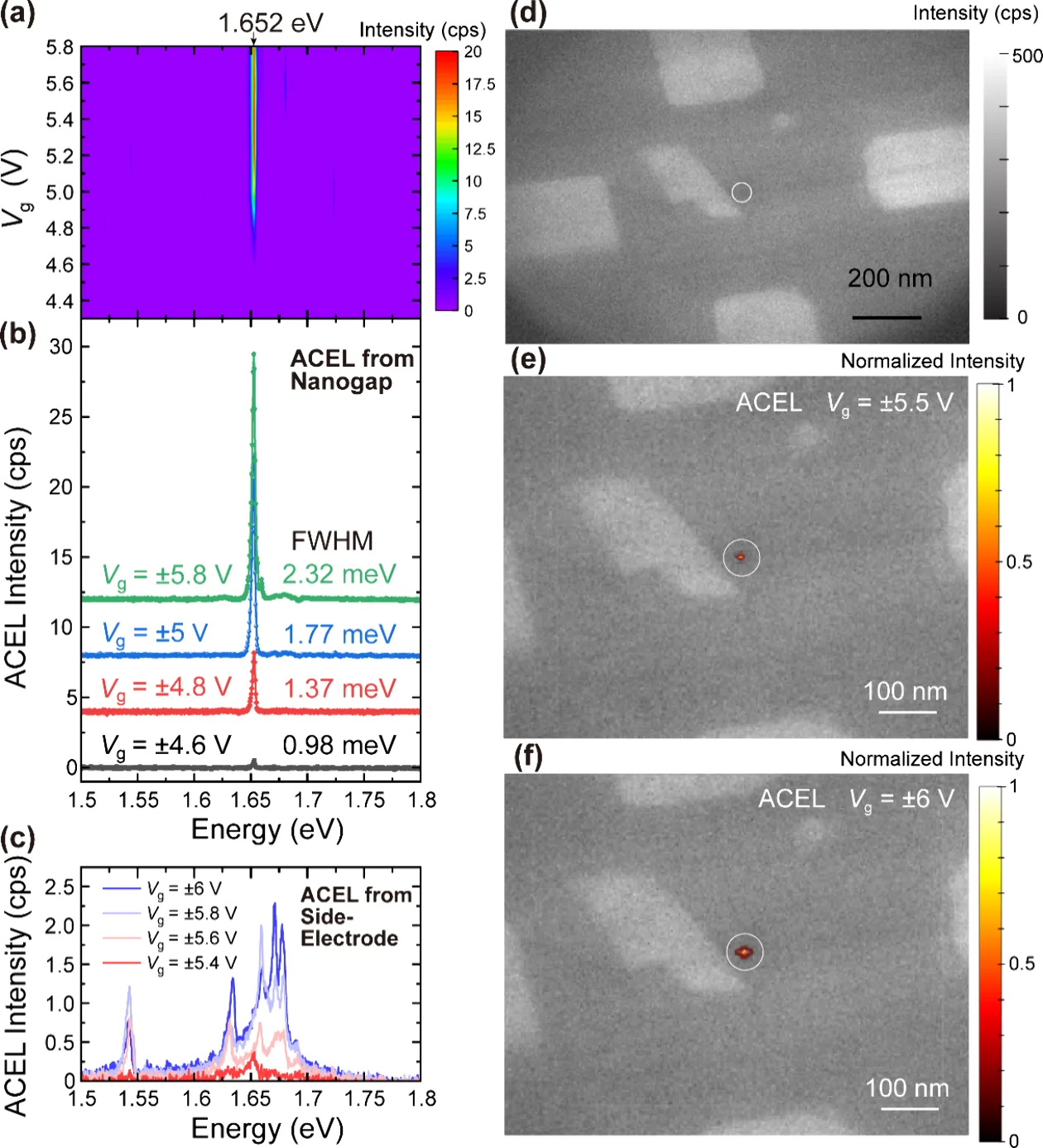
Voltage-dependent emission and spatial localization of nanogap ACEL. (a) Two-dimensional contour map of ACEL intensity from nanogap as a function of emission energy and *V*_g_ for the same device shown in Figure 1e. A dominant emission peak centered at ∼1.652 eV first becomes detectable at *V*_g_ = ±4.6 V and remains spectrally stable up to *V*_g_ = ±5.8 V. (b) Representative ACEL spectra from the sub-5 nm nanogap at different *V*_g_ (±4.6 to ±5.8 V, 10 MHz, 3.5 K), showing a dominant narrow emission peak with increasing intensity while the peak energy remains invariant, and a line width ranging from ∼0.98 to 2.32 meV; these values include the instrumental response and were not deconvolved. (c) ACEL spectra measured from the wider side-electrode gap (∼35 nm) at higher voltages (*V*_g_ ∼ ±5.4 to ±6.0 V, 10 MHz, 3.5 K). In contrast to the nanogap emission, multiple broadened peaks appear over a wider energy range, indicating the absence of strong spatial confinement. (d) Optical microscopy image of the device with the nanogap region marked (circle). (e, f) Overlaid spatial maps of ACEL intensity acquired at *V*_g_ = ±5.5 V (e) and ±6.0 V (f) (10 MHz, 3.5 K), showing a point-like emission localized at the nanogap center. The emission intensity increases with voltage while remaining spatially confined.

As shown in Figure 2b, increasing *V*_g_ leads to a steady increase in emission intensity. The apparent fitted fwhm values, which include the unresolved instrumental response, are 0.98, 1.37, 1.77, 1.83, and 2.32 meV at ± 4.6, ± 4.8, ± 5.0, ± 5.4, and ± 5.8 V, respectively. No instrumental-response deconvolution was performed. The measured signal-to-background ratios (SBRs) ranged from 24.1 to 51.2 at voltage amplitudes from ± 4.8 to ± 5.8 V (Table S1). This invariance of the peak energy further supports the conclusion that the emission originates from a discrete, localized state rather than band-edge recombination.

This reproducibility is further confirmed in additional devices (Figure S4), each of which exhibits narrow ACEL despite device-to-device variations in emission energy. The ACEL peak energies and corresponding Lorentzian fwhm values for Devices A-E are 1.6879 eV (3.45 meV), 1.6604 eV (1.68 meV), 1.6010 eV (2.08 meV), 1.6661 eV (2.48 meV), and 1.6828 eV (4.28 meV), respectively. Importantly, these devices also show multipeak PL spectra, reflecting the behavior of the main device shown in Figures 1 and 2. At higher driving voltages, additional discrete emission peaks emerge sequentially at different energies, as shown in Figure S4. Across 49 devices exhibiting measurable current–voltage responses and detectable WSe_2_ PL, 15 showed sharp-feature ACEL, 13 showed broad/mixed ACEL, and 21 showed no detectable ACEL, while the 14 monolayer sharp-ACEL devices exhibited a median Lorentzian fwhm of 2.07 meV (Tables S2 and S3).

To examine the effect of gap separation on ACEL, we performed a control experiment on the same device using the adjacent side-electrode channel (∼35 nm gap, Figure 1c). The emission characteristics at the side-electrode show multiple broad, overlapping peaks across a wider energy range, from 1.63 to 1.69 eV, with an additional peak at 1.542 eV (Figure 2c), which differ notably from those of the sub-5 nm gap (Figure 2b). The first detected ACEL voltage increased to ± 5.4 V, at which the peak intensity remains below 1 count per second (cps).

In contrast, high-resolution spatial mapping locates the origin of the emission. The open circle in the optical microscope image (Figure 2d) marks the center of the nanogap. The corresponding ACEL intensity maps acquired at *V*_g_ = ± 5.5 V and ± 6.0 V (Figure 2e and f) reveal a point-like emission localized at this position, which brightens with increasing voltage and remains spatially confined at the nanogap. At higher voltage amplitudes of ±6.5 and ±7.0 V, the emission profile becomes spatially extended and develops an elliptical shape elongated along the nanowire direction (Figure S5). This evolution is consistent with carrier redistribution from the nanogap-centered region into neighboring emitting states that become accessible at higher injection levels. The observed anisotropy may reflect the local electrode geometry and strain distribution surrounding the nanogap.

Figure 3 illustrates the proposed mechanism of nanogap ACEL. Figure 3a summarizes the square-wave gate excitation sequence used to drive carrier injection, trapping, recombination, and extraction. The band alignment at *V*_g_ = 0 is based on the energy levels of WSe_2_ and Au, with *E*_c_ = 3.7 eV, *E*_v_ = 5.44 eV, and an intrinsic Fermi level *E*_f_ ∼ 4.57 eV for WSe_2_ at 3.5 K, and *E*_f_ = 5.1 eV for Au (Figure 3b).^10,38^ The sub-5 nm channel length causes the band-bending regions from both Au/Pt–WSe_2_ contacts to merge across the channel, producing a strongly nonuniform potential landscape across the nanogap. This geometry concentrates the effective carrier–defect interaction within a nanoscale volume.^9,10,38–40^

**Figure 3:**
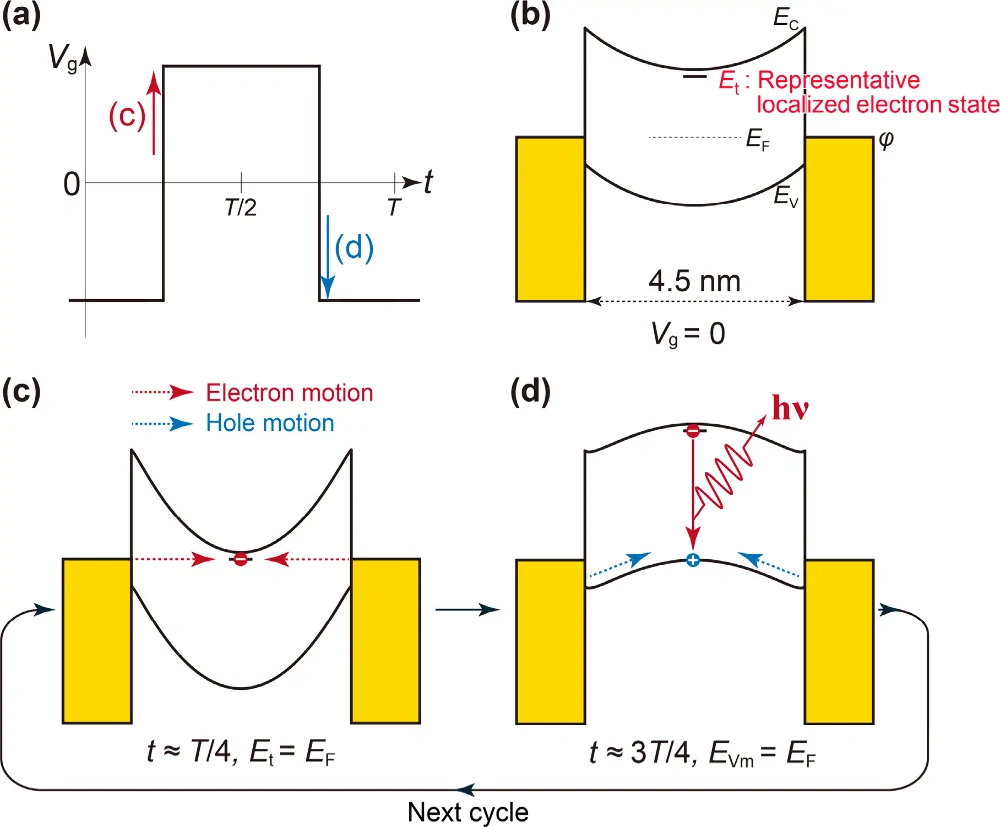
Proposed mechanism of localized-state-mediated ACEL in the sub-5 nm nanogap. The position and number of localized states are schematic and do not indicate a unique trap. Red and blue dashed arrows indicate the proposed directions of electron and hole motion, respectively. The return arrow from (d) to (c) denotes the subsequent excitation cycle. (a) Schematic of the applied square-wave *V*_g_ and corresponding phases within one cycle. (b) Schematic energy band diagram of the nanogap at *V*_g_ = 0, showing a representative localized electron state (*E*_t_) positioned below the conduction band edge within the nanogap region (∼4.5 nm). (c) During the positive half-cycle (t ≈ *T*/4), field-induced band modulation drives electrons from the grounded Au/Pt electrodes toward the nanogap region, where localized electron states are populated. (d) During the negative half-cycle (t ≈ 3*T*/4), the reversed band modulation promotes hole injection and enables radiative recombination through a representative localized emitting state, producing a photon with energy *hν*.

To quantitatively evaluate the electric-field distribution near the sub-5 nm nanogap, we performed a three-dimensional finite-element electrostatic simulation using the experimentally relevant device geometry (Figure S8). With both Pt/Au electrodes grounded and the Si back gate biased at *V*_g_ = 5.4 V, the calculated field in WSe_2_ is strongly localized near the curved Au electrode boundaries adjacent to the 4.5 nm gap. The in-plane electric-field component reaches approximately 0.037 V/nm near the Au/WSe_2_ boundaries, while the total electric-field magnitude reaches approximately 0.044 V/nm. At the geometrical center of the gap, the in-plane component approaches zero because the lateral field contributions from the two symmetrically grounded electrodes cancel. These results demonstrate that the regions of enhanced electric field are spatially confined near the two Au/WSe_2_ contact boundaries bordering the nanogap rather than exactly at the gap center.

Transfer of the WSe_2_ monolayer onto the spherical Au nanoparticles produces interfacial adhesion and strain in the portion of the monolayer bridging the two contacts (Figure S6), aligning with reported Au–WSe_2_ interfacial interactions.^41,42^ To explore the resulting strain distribution, we developed a qualitative atomistic model of a WSe_2_ ribbon bridging two Au hemispheres using a universal machine-learning interatomic potential. The calculated *ε*_*xx*_ distribution is highly nonuniform, with tensile regions approaching approximately 0.5–1.0% above the Au hemispheres, where the monolayer bends around the spherical surfaces, and neighboring compressive regions extending across the intervening nanogap region (Figure S7). Although the precise strain distribution depends on the transfer process, thermal history, and interfacial adhesion, the model supports the formation of rapidly varying nanoscale strain gradients induced by the curved Au electrode topography.

The calculated nonuniform strain landscape indicates that the spherical Au/Pt electrodes may modify the local electronic environment near the nanogap. However, the present structural and optical data do not uniquely determine whether strain is responsible for the formation of the emitting localized state. States that spatially overlap regions of enhanced electric field near the curved Au boundaries bordering the nanogap may contribute preferentially to ACEL. The ACEL line at 1.652 eV is red-shifted by approximately 88 meV from the neutral exciton emission of the WSe_2_ monolayer near 1.74 eV, supporting its assignment to localized state emission.

Under square-wave excitation, periodic modulation of the band alignment enables phase-dependent carrier injection and recombination in the sub-5 nm nanogap. During the positive half-cycle, downward conduction-band bending allows electrons to enter regions of enhanced electric field near the curved Au boundaries bordering the nanogap and become trapped in localized states below the conduction-band edge (Figure 3c). Reversal of the gate polarity produces the opposite band bending, promoting hole injection and radiative recombination with the trapped electrons (Figure 3d). At 10 MHz (T = 100 ns), this mechanism requires the electron detrapping time to exceed the half-period of approximately 50 ns, allowing electrons captured during one phase to persist into the subsequent phase. The injected holes are transient and are extracted toward the metallic electrodes upon the next polarity reversal.

The waveform dependence further supports the importance of rapid voltage transients. A sinusoidal voltage of the same amplitude and frequency produced no detectable ACEL (Figure S9), whereas square-wave excitation generated a pronounced emission signal. Compared with a sinusoidal waveform, a square wave exhibits steeper voltage transitions and remains near its maximum amplitude for a larger fraction of each half-cycle. These characteristics favor transient carrier injection, trapping, and subsequent recombination, in agreement with previous ACEL studies.^31–33^

Complementary measurements on two additional devices demonstrate that the ACEL spectrum is sensitive to both excitation frequency and voltage-transition time. In one device, increasing the square-wave frequency from 1 to 3 MHz increased the background-referenced peak height near 1.64 eV from approximately 1 to 6 cps and reduced the apparent fwhm of the composite emission envelope from approximately 47 to 14 meV (Figure S10). In another device, shortening the voltage-transition time from 20 to 8.4 ns produced a progressively more pronounced and spectrally better-resolved emission feature (Figure S11). These trends corroborate the importance of high frequency and rapid voltage transitions in promoting transient carrier injection and altering carrier redistribution among localized states. Because the measurements were obtained from different devices, they are interpreted independently rather than as a single quantitative dependence.

Control devices further clarify the structural requirements for narrow-line-width ACEL. Devices with a 2 μm electrode separation exhibited only broad emission (Figure S12), whereas insertion of a SiO_*x*_ spacer between WSe_2_ and the electrodes produced weak, broad emission (Figure S13). Consistent with the SiO_*x*_-spacer control experiments, three-dimensional electrostatic simulations show that the 1.5 nm SiO_*x*_ layer reduces the peak electric field along WSe_2_ from approximately 0.044 to 0.029 V/nm, corresponding to a reduction of approximately 34% (Figure S13j). The reduced field is consistent with weakened electrical coupling between the Pt/Au electrodes and WSe_2_ and with the weak and broad ACEL observed in the spacer-containing devices. Coupling WSe_2_ to an extended Au/Pt nanowire also generated multiple broadened peaks, indicating the activation of spatially distributed emitting states (Figure S14). Thus, neither an extended channel nor a spatially distributed WSe_2_–electrode interface reproduced the narrow emission obtained from the sub-5 nm nanogap.

Together, the electrical-driving and structural-control experiments support a localized-state-mediated ACEL mechanism in which rapid polarity reversal promotes carrier injection, while the sub-5 nm junction spatially confines the electrically accessed localized states. We next examine the spectral characteristics of this localized emission through the line shape analysis presented in Figure 4.

**Figure 4:**
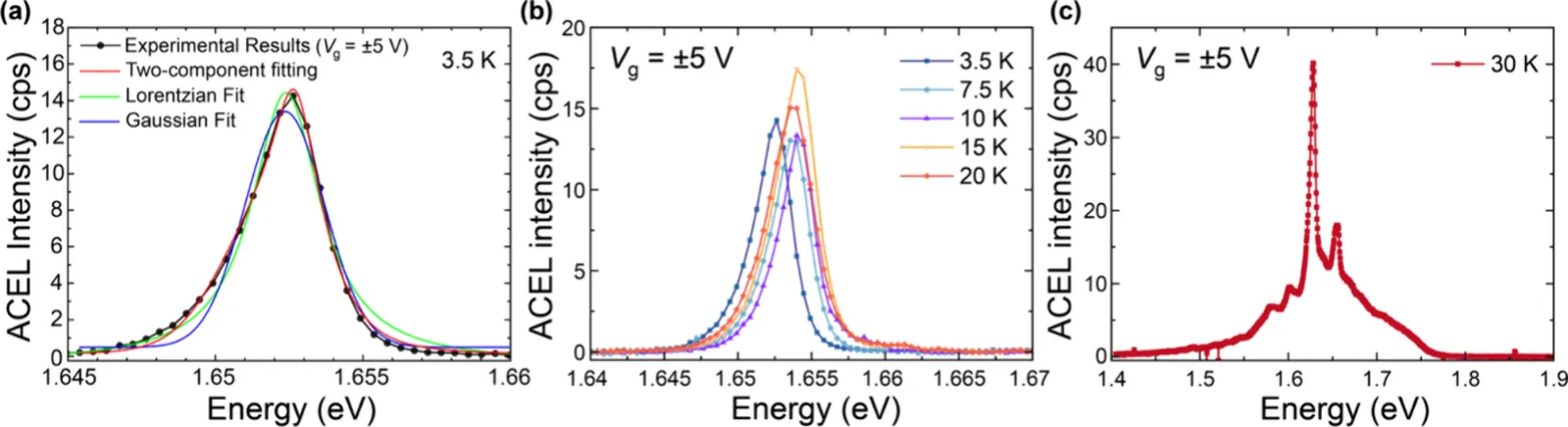
Spectral decomposition and temperature-dependent evolution of ACEL at ± 5 V. (a) ACEL spectrum acquired at 3.5 K, showing an asymmetric line shape with a broader low-energy tail. The spectrum is well reproduced by an empirical additive two-component model (red curve; *R* ^2^ = 0.996), comprising a Lorentzian component phenomenologically assigned to the dominant zero-phonon line and an independently centered, red-shifted Gaussian component representing a broader phonon-assisted contribution. The fitted Lorentzian fwhm is 1.97 meV. The green and blue curves show separate fits using a Lorentzian function and a Gaussian function, respectively. (b) Temperature-dependent ACEL spectra acquired from the same device between 3.5 and 20 K. Consistent two-component fitting yields Lorentzian fwhm values of 1.98, 1.95, 2.19, 2.23, and 2.67 meV at 3.5, 7.5, 10, 15, and 20 K, respectively. (c) ACEL spectrum acquired at 30 K over an extended energy range. The substantially altered spectrum exhibits multiple emission features and a broad underlying background, indicating a pronounced temperature-dependent spectral reconfiguration rather than simple quenching of the low-temperature emission feature.

The ACEL spectrum at 3.5 K shows a noticeable asymmetry, with a broader low-energy side compared to the high-energy side (Figure 4a). The main peak and high-energy flank are described primarily by the Lorentzian component, whereas the broader low-energy shoulder is captured by an independently centered, red-shifted Gaussian component. Accordingly, the spectrum is fitted using an empirical additive two-component model consisting of a Lorentzian and a Gaussian component with a red-shifted center^43–45^ (Figure 4a, red), which reproduces the ACEL spectrum with high fidelity (*R* ^2^ = 0.996). The Lorentzian component, centered at 1.6527 eV with a fwhm of 1.97 meV, is phenomenologically assigned to the dominant zero-phonon line (ZPL), whereas the broader Gaussian component represents a phonon-sideband (PSB) contribution. The detailed analysis is described in Supplementary Note 1.

Within this empirical decomposition, the two components are interpreted phenomenologically as having different possible physical contributions. The Lorentzian component is assigned to a dominant ZPL-like contribution, whereas the red-shifted Gaussian component is assigned to a broader phonon-sideband-like contribution. These assignments are based on the spectral decomposition and do not constitute independent identification of the underlying physical processes.

Within the adopted phenomenological decomposition and Debye–Waller approximation, the fitted area ratio of ∼ 1.25 corresponds to an effective Huang–Rhys factor of *S* ∼ 0.59. This model-dependent value suggests relatively weak electron–phonon coupling,^46^ but it should not be interpreted as an independently determined intrinsic material parameter. The apparent fitted Lorentzian fwhm is 1.97 meV. Because the instrumental response was not independently calibrated or deconvolved, this value should not be interpreted as the intrinsic line width or used to quantify homogeneous broadening or spectral diffusion arising from local charge fluctuations.^47^ Nevertheless, the apparent fitted line width is comparable in magnitude to line widths reported for localized emitters in WSe_2_.^9,10,38,39^ The observed spectrum features a dominant narrow emission line accompanied by a weaker red-shifted sideband, consistent with a dominant localized emission feature. Photon-correlation measurements will be required to determine whether the emission exhibits nonclassical photon statistics.

The temperature dependence was examined using the same device operated at *V*_g_ = ±5 V. The Lorentzian fwhm remains approximately 1.95–1.98 meV between 3.5 and 7.5 K, increases to 2.19 and 2.23 meV at 10 and 15 K, respectively, and reaches 2.67 meV at 20 K (Figure 4b). Over the same temperature range, the Lorentzian center shifts from 1.6527 eV at 3.5 K to 1.6543 eV at 15 K, followed by a slight shift to 1.6539 eV at 20 K. These results show that the localized emission feature persists up to 20 K while undergoing moderate spectral broadening and a small variation in emission energy.

At 30 K, the spectrum changes substantially, displaying increased intensity, multiple emission features, and a broad underlying background over an extended energy range (Figure 4c). This spectral reconfiguration is inconsistent with simple thermal quenching of a single emitting state and may reflect temperature-dependent changes in carrier injection or redistribution among multiple localized states. Accordingly, no single activation or localization energy is extracted from these measurements.

The material specificity of this emission was examined by substituting WSe_2_ with other TMDs. The nanogap architecture also produces narrow-line-width ACEL in bilayer WSe_2_ (Figure S15), aligning with reports of localized emission in both monolayer and bilayer WSe_2_.^48,49^ Other TMDs exhibit qualitatively different behavior. In contrast, monolayer MoS_2_ devices (Figure S16) show no detectable ACEL under the present nanogap-device and excitation conditions. This result does not indicate that monolayer MoS_2_ is intrinsically incapable of ACEL, because transient-mode ACEL has previously been demonstrated in monolayer MoS_2_.^31^ The absence of detectable emission here may instead reflect sample- and device-dependent carrier dynamics. In particular, the high intrinsic electron density of as-exfoliated MoS_2_ may favor trion formation, while defect-assisted nonradiative pathways may further reduce the radiative recombination yield in the present devices. Monolayer WS_2_ devices (Figure S17) exhibit ACEL only as a broad emission band spanning 1.56–1.90 eV, without spectrally resolved features, reflecting a heterogeneous defect landscape with overlapping contributions from multiple defect species, even under optimized strain engineering conditions.^50^ These results demonstrate that the narrow-line-width ACEL observed here is not a generic consequence of the sub-5 nm nanogap geometry, but instead likely reflects material- and device-dependent localized-state and carrier-injection characteristics in monolayer WSe_2_.

In summary, the lithographically defined sub-5 nm spherical Au/Pt nanogap architecture produces spectrally isolated narrow-line-width ACEL from monolayer WSe_2_ with an apparent fitted line width of approximately 2 meV under square-wave AC gate excitation. The resulting spectrally isolated and high-SBR ACEL, in stark contrast to the broad, multipeak PL, suggests a spatial confinement of emitting states and phase-selective carrier injection. Waveform-dependent measurements and structural control devices further support a localized-state-mediated mechanism promoted by rapid voltage transitions and nanoscale spatial confinement. Structural modeling indicates a nonuniform strain landscape near the curved electrodes; however, the present results do not uniquely establish strain as the microscopic origin of the emitting state. Lineshape analysis reveals a dominant ZPL-like contribution accompanied by a weaker red-shifted PSB-like contribution, consistent with localized-state emission and relatively weak electron–phonon coupling. These results establish a planar strategy for narrow-line-width, electrically driven localized emission in monolayer WSe_2_. More broadly, lithographically defined nanogaps could provide a general platform for electrically addressing nanoscale emitters, including localized states in 2D semiconductors, semiconductor quantum dots, and other low-dimensional material systems. Future photon-correlation measurements will offer further insight into the nature of the localized emitting states under strong nanoscale field confinement.

## Methods

### Device Fabrication

The initial ∼15 nm Pt nanogap electrodes were patterned on heavily doped silicon substrates with a 50 nm thermally grown SiO_2_ layer (15 × 15 mm^2^) (Figure 1a). A Pt (10 nm)/Ti (3 nm) structure was defined by electron-beam lithography (EBL; ELS-7500EX, Elionix, Japan), followed by a standard lift-off process, yielding a gap separation of ∼ 15 nm and a line width of ∼12 nm.

Prior to gap narrowing, the electrodes were cleaned by ultraviolet (UV) ozone treatment. Gap reduction was achieved using a previously established electroless gold plating (ELGP) technique.^34–37^ The plating solution was prepared by dissolving a high-purity Au sheet (99.99%) in iodine tincture under ultrasonic agitation, followed by *L*(+)-ascorbic acid to saturation as a reducing agent. Immersion of the Pt nanogap electrodes into this solution resulted in the selective growth of heteroepitaxial spherical Au nanoparticles on the Pt surfaces, reducing the gap separation to the sub-5 nm regime (e.g., 4.5 nm). The process yielded ∼90% of devices with gap separations below 5 nm. Further details are provided in Figure S1.

WSe_2_ (HQ graphene) was mechanically exfoliated from bulk crystals onto 50 nm SiO_2_/Si substrates using adhesive tape. Selected monolayers were transferred onto prepatterned nanogap electrodes on SiO_2_/Si substrates using a dry transfer technique described in Figure S2.

### Photoluminescence and AC Electroluminescence Measurements

Unless otherwise specified, optical and electrical measurements were performed at 3.5 K in a closed-cycle optical cryostat (Cryostation s200, Montana Instruments) under high vacuum. The WSe_2_ nanogap devices were mounted on a sample holder and electrically connected through wire bonds formed using silver paste.

Both photoluminescence (PL) and alternating-current electroluminescence (ACEL) signals were measured using a custom-built micro-PL spectroscopy setup. Emission from the nanogap was collected through a 50× objective lens (numerical aperture, NA = 0.42).

For PL measurements, a continuous-wave 532 nm laser was used as the excitation source. The emitted photons were passed through a 550 nm dielectric long-pass filter to suppress excitation laser scattering.

For ACEL excitation, a 10 MHz bipolar square-wave voltage was generated by an arbitrary waveform generator (Agilent 33522A) and applied to the global silicon back gate, while the nanogap electrodes were grounded. Hardware control and synchronized data acquisition were implemented using a custom Python routine based on the open-source ScopeFoundry platform.

The emission signals were dispersed by a high-resolution spectrograph (Shamrock 500i, Andor) equipped with a 150 grooves/mm grating, and detected using a silicon charge-coupled device (CCD; Newton 971, Andor). Spatial emission images for both PL and ACEL were acquired using an electron-multiplying CCD (EMCCD; Luca, Andor). The dark-count background of the CCD was measured and subtracted prior to each spectral acquisition. The ACEL spectra were acquired using the same optical collection and spectroscopic measurement configuration, including the same entrance-slit setting, as in our previous ACEL studies.^31,51^ No independent calibration of the instrumental spectral response under the present acquisition conditions or instrumental-response deconvolution was performed. Accordingly, all reported line widths are apparent fitted line widths that include the instrumental response and should not be interpreted as deconvolved intrinsic line widths.

### Empirical Two-Component Spectral Fitting

Spectral fitting was performed in OriginPro and Python using an empirical additive two-component model consisting of a Lorentzian main peak and an independently centered, red-shifted Gaussian component. The fit shown in Figure 4a was performed in OriginPro, whereas the temperature-dependent linewidths reported for Figure 4b were obtained from independent fits in Python. Following previous analyses of localized WSe_2_ emission, the components were assigned phenomenologically to the dominant zero-phonon-like emission and a broader phonon-sideband-like contribution, respectively.^44,45^ The reported line width corresponds to the fwhm of the Lorentzian component. No deconvolution of the instrumental response was performed.

## Supplementary Material



## Notes

Additional experimental details for HS-Au/Pt nanogap fabrication, monolayer WSe_2_ dry transfer, and device characterization; signal-to-background analysis; device reproducibility and statistics; strain and electrostatic simulations; ACEL measurements under varied voltage waveform, frequency, and transition-time conditions; control experiments using micrometer-gap, spacer, nanowire, bilayer WSe_2_, monolayer MoS_2_, and monolayer WS_2_ devices; spectral decomposition and effective Huang–Rhys factor estimation (PDF)
