# Supplementary material for: Narrow-Line-Width Electroluminescence in Monolayer WSe2 Enabled by a Sub‑5 nm Au/Pt Nanogap
Source: Nano Lett. 2026 Oct 3;26(40):13862–70. doi: 10.1021/acs.nanolett.6c03133 (PMC13674333; doi:10.1021/acs.nanolett.6c03133)
Supplement: Supplementary file 1 [file nl6c03133_si_001.pdf]

Supporting Information

Narrow-Line-Width Electroluminescence in Monolayer WSe_2 Enabled by a Sub-5 nm Au/Pt Nanogap

Shurong Miao,[1,2,3,†] I. K. M. Reaz Rahman,[1,3,†] Aditya Nirmale,[2] Inha Kim,[1,3] Dorottya Urmossy,[1,3] Thomas P. Darlington,[4] Harishankar Jayakumar,[4] Seiichiro Izawa,[2] Archana Raja,[4,6] Daryl C. Chrzan,[3,5] Yutaka Majima,[2,*] and Ali Javey,[1,3,6,*]

[1] Electrical Engineering and Computer Sciences, University of California, Berkeley, California 94720, United States;

[2] Materials and Structures Laboratory, Institute of Science Tokyo, Yokohama 2268503, Japan;

[3] Materials Sciences Division, Lawrence Berkeley National Laboratory, Berkeley, California 94720, United States;

[4] The Molecular Foundry, Lawrence Berkeley National Laboratory, Berkeley, CA 94720, USA

[5] Department of Materials Science and Engineering, University of California, Berkeley, Berkeley, CA 94720, USA

[6] Kavli Energy NanoScience Institute at the University of California, Berkeley, Berkeley, California 94720, United States.

† These authors contributed equally: Shurong Miao and I. K. M. Reaz Rahman.

[*]Corresponding authors: Yutaka Majima and Ali Javey.

Email: majima@msl.titech.ac.jp; ajavey@berkeley.edu

Supporting Information

Table of Contents

Figure S1	HS-Au/Pt nanogap fabrication and structural characterization	3
Figure S2	Dry transfer of monolayer WSe_2 onto the nanogap	5
Figure S3	Room-temperature DC transport characteristics	6
Table S1	Fixed-window signal-to-background ratio analysis	9
Figure S4	Representative device-to-device spectral reproducibility	10
Table S2	Outcome distribution of all 49 measured devices	13
Table S3	Spectral and voltage statistics for 14 monolayer sharp-ACEL devices	14
Figure S5	Spatial ACEL profiles at elevated voltage amplitudes	16
Figure S6	Schematic of conformal attachment and strain formation	17
Figure S7	Atomistic modeling of strain in the WSe_2 nanogap	19
Figure S8	WSe_2 nanogap electrostatic simulation	21
Figure S9	ACEL response under sinusoidal excitation	24
Figure S10	Frequency-dependent ACEL spectra from 1 to 3 MHz	25
Figure S11	Voltage-transition-time-dependent ACEL spectra	27
Figure S12	Optical and electrical characterization of 2 μm control devices	29
Figure S13	Effect of a 1.5 nm SiO_x interfacial spacer	31
Note 1	Lorentzian-Gaussian decomposition and effective Huang-Rhys factor	34
Figure S14	Multi-defect 1D nanowire control device	36
Figure S15	Bilayer WSe_2 nanogap device	38
Figure S16	Monolayer MoS_2 nanogap control devices	40
Figure S17	Monolayer WS_2 nanogap control device	41
References	Supporting Information references	42

HS-Au/Pt nanogap fabrication and structural characterization

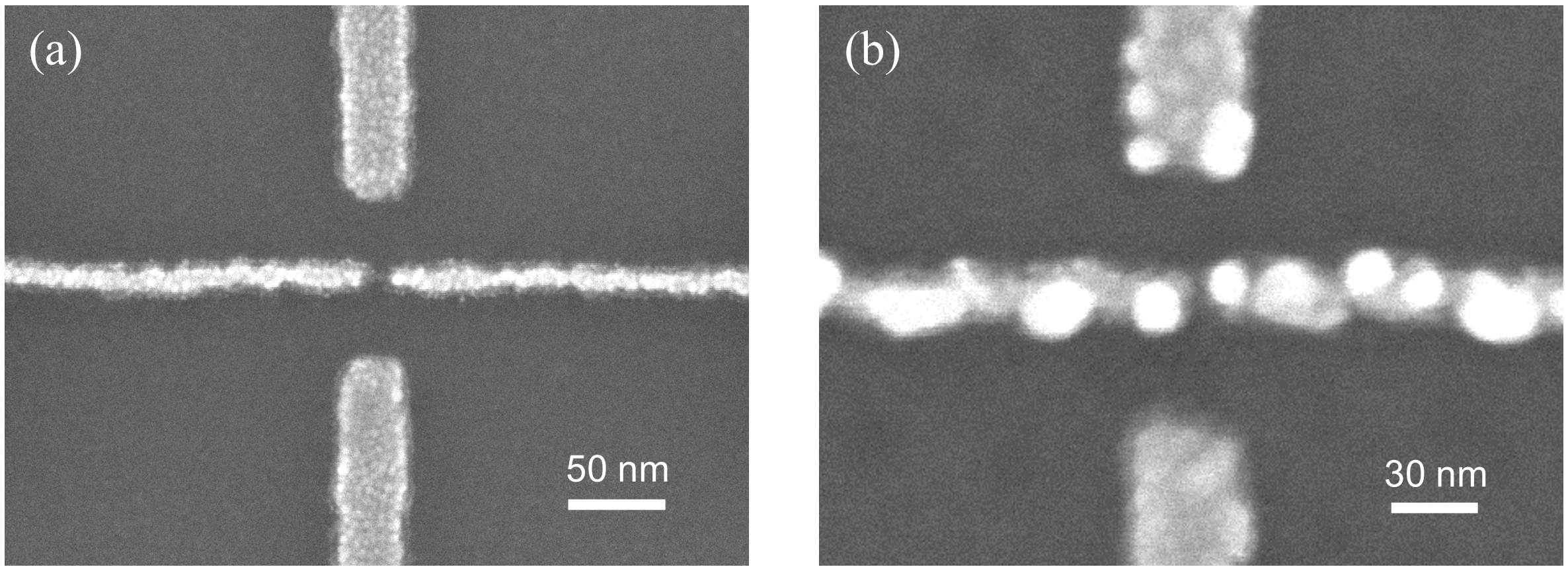

Figure S1. Structural characterization of the heteroepitaxial spherical (HS)-Au/Pt nanogap. SEM images of (a) the initial Pt nanogap electrodes with side electrodes defined by electron-beam lithography (EBL) and (b) the HS-Au/Pt nanogap electrodes after electroless gold plating (ELGP).

The heteroepitaxial spherical Au/Pt (HS-Au/Pt) nanogap architecture with side electrodes was fabricated using high-resolution top-down lithography and bottom-up heteroepitaxial growth. Heavily doped silicon wafers featuring a 50-nm-thick thermally grown SiO_2 layer were diced into 15 mm × 15 mm substrates. The substrates were cleaned in acetone and ethanol (2 min each, repeated twice), followed by an ultraviolet/ozone (UV/O_3) treatment (3 min O_2 purging, 12 min UV irradiation, and 3 min N_2 purging).

The initial Pt nanogap structures were defined by electron-beam lithography (EBL; ELS-7500EX, ELIONIX, Japan) using a positive-tone electron-beam resist (ZEP-520A, Zeon, Japan). After development, Pt/Ti (10 nm/3 nm) was deposited via thermal evaporation, followed by lift-off in ZDMAC to form the initial Pt nanogap electrodes.

To enable large-area electrical connections, contact pads were fabricated by photolithography. A positive photoresist (S1818, DuPont, Japan) was spin-coated and patterned using a mask aligner (MA-20, MIKASA, Japan). A thicker Pt/Ti layer (40 nm/5 nm) was then deposited by thermal evaporation, followed by lift-off in acetone to complete the Pt device structure (Figure S1a).

To reduce the initial lithographic gap down to the sub-5 nm regime, electroless gold plating (ELGP) was employed, enabling heteroepitaxial spherical (HS) growth of Au on Pt electrodes and the formation of HS-Au/Pt nanogaps (Figure S1b), following procedures described in our previous reports.[1-4]

Dry transfer of monolayer WSe_2 onto the nanogap

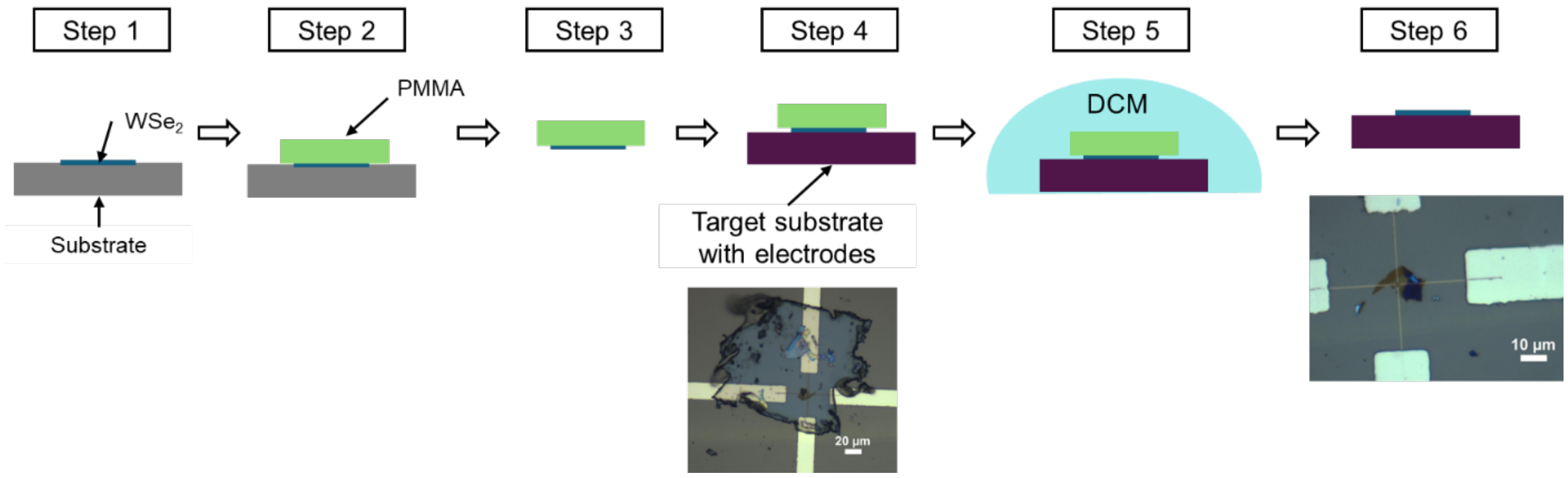

Figure S2. Deterministic dry-transfer process for integrating monolayer WSe_2 onto HS-Au/Pt nanogap electrodes. Schematic illustration of the PMMA-assisted dry-transfer procedure, including monolayer identification, PMMA pickup, alignment to the nanogap electrodes, transfer, and PMMA removal. Optical microscope images show the device immediately after transfer (left) and after PMMA removal (right).

WSe_2 flakes (HQ Graphene) were mechanically exfoliated from bulk synthetic crystals using adhesive tape (3M) and transferred onto a viscoelastic polydimethylsiloxane (PDMS) stamp mounted on a glass slide. The PDMS stamp was inverted and brought into contact with a clean SiO_2/Si substrate (50 nm oxide) to transfer the exfoliated flakes. The substrate was systematically inspected under a bright-field optical microscope to identify monolayer WSe_2 regions based on their characteristic optical contrast.

The SiO_2/Si substrate carrying the identified WSe_2 monolayer was mounted onto a multi-axis micromanipulator within a custom-built transfer system. A thin polymethyl methacrylate (PMMA) layer was deposited onto the target WSe_2 flake and annealed at 180 °C for 1 min to ensure conformal contact. The PMMA/monolayer stack was picked up and aligned to the center of the pre-patterned HS-Au/Pt cross-nanogap electrodes. A second annealing step at 180 °C for 3 min promoted conformal contact between the WSe_2 monolayer and the nanogap electrodes, minimizing interfacial voids. Finally, the PMMA was removed by immersion in dichloromethane (DCM) for 1 min, leaving the WSe_2 monolayer directly integrated onto the sub-5 nm nanogap for subsequent optoelectronic characterization.

Room-temperature DC transport characteristics

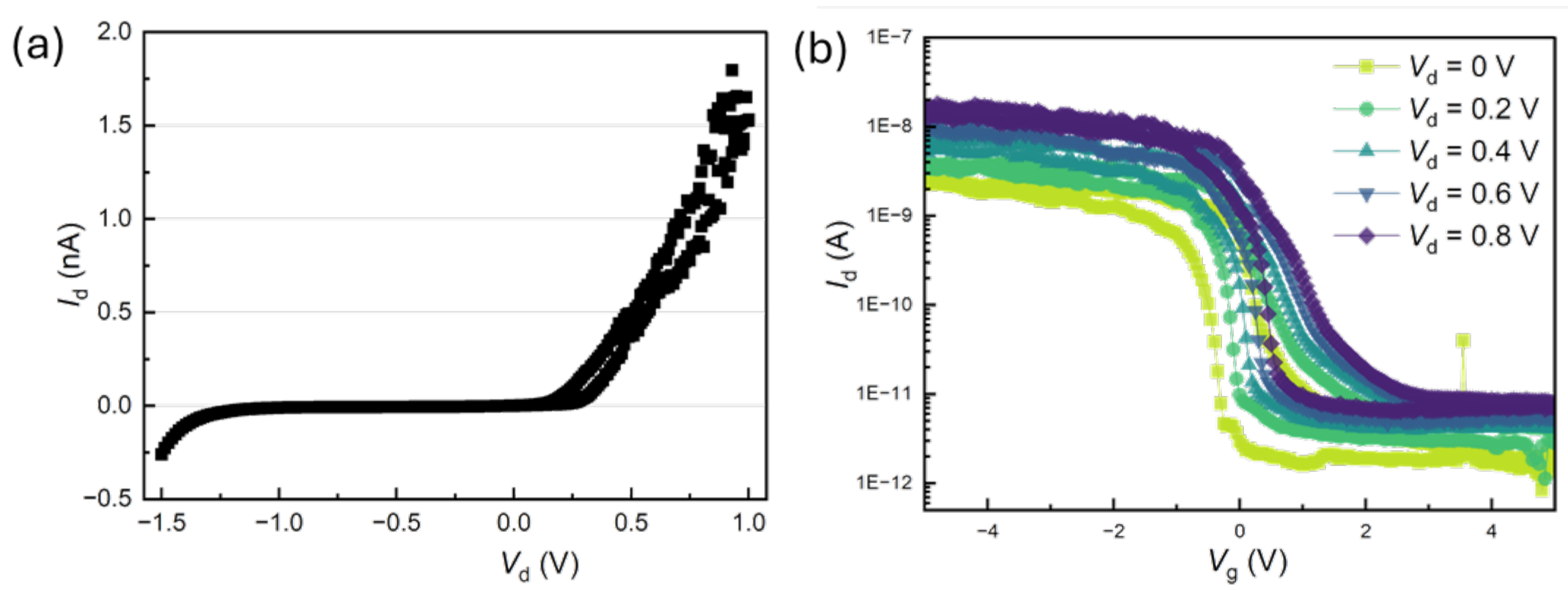

Figure S3. Room-temperature DC transport characteristics of the monolayer WSe_2 nanogap devices. (a) Output characteristics ($I_d - V_d$) measured at $V_g = 0$ V. (b) Transfer characteristics ($I_d - V_g$) of the same device measured at $V_d = 0 - 0.8$ V on a semi-logarithmic scale. The device exhibits *p*-type transport behavior.

To evaluate the electrical quality of the integrated WSe_2 devices, direct-current (DC) transport measurements were performed at room temperature using a probe station equipped with a semiconductor parameter analyzer (Agilent 4156C).

The output ($I_d - V_d$) and transfer ($I_d - V_g$) characteristics shown in Figure S3 confirm successful electrical integration of the monolayer WSe_2 with the HS-Au/Pt electrodes. The transfer characteristics exhibit clear *p*-type transport behavior.

Fixed-window analysis of the ACEL signal-to-background ratio

The ACEL spectra were acquired using the same optical collection and spectroscopic measurement configuration, including the same entrance-slit setting, as in our previous ACEL studies.[5,6] No independent calibration of the instrumental spectral response under the present acquisition conditions or instrumental-response deconvolution was performed. Each dark-subtracted ACEL spectrum was analyzed using three fixed energy intervals: a peak window of 1.642–1.662 eV, a left background window of 1.620–1.640 eV, and a right background window of 1.664–1.684 eV. These intervals were applied without adjustment to all gate-voltage amplitudes.

The mean intensities within the left and right background windows are denoted as $\bar{I}_L$ and $\bar{I}_R$, respectively. The off-peak background level was defined as

$$\bar{I}_{bg} = \frac{\bar{I}_L + \bar{I}_R}{2}$$

The integrated peak area, A_{peak}, was obtained by trapezoidal integration over the measured energy coordinates within 1.642–1.662 eV. The background area expected over the same spectral width was calculated as

$$A_{bg} = \bar{I}_{bg} \Delta E_{peak}$$

where ΔE_{peak} is the actual sampled energy span of the peak window (0.01953 eV). The background-corrected peak area was then determined from

$$A_{net} = A_{peak} - A_{bg}$$

and the SBR was calculated as

$$SBR = \frac{A_{net}}{A_{bg}}$$

No smoothing, normalization, clipping of negative values, or additional baseline correction was performed. For the spectra acquired at gate-voltage amplitudes of 4.8–5.8 V, the residual background was positive, and the resulting SBR values ranged from 24.1 to 51.2 (Table S1). At 4.6 V, the mean off-peak background was −0.025 cps after dark subtraction. Because this value

gives a nonpositive background area, the corresponding SBR was reported as not determined rather than assigning an artificial value.

Table S1. Quantitative signal-to-background (SBR) analysis of the ACEL spectra.

V_g (V)	Mean off-peak background (cps)	Net peak area (cps·eV)	Measured SBR
±4.6	−0.025	0.00137	Not determined[a]
±4.8	0.028	0.01386	25.0
±5.0	0.050	0.05027	51.2
±5.2	0.083	0.06419	39.4
±5.4	0.081	0.07079	44.5
±5.6	0.168	0.07907	24.1
±5.8	0.127	0.07191	29.0

[a]The mean residual background at 4.6 V is negative after subtraction of the independently measured mean dark spectrum. A direct SBR is not defined.

Representative device-to-device spectral reproducibility

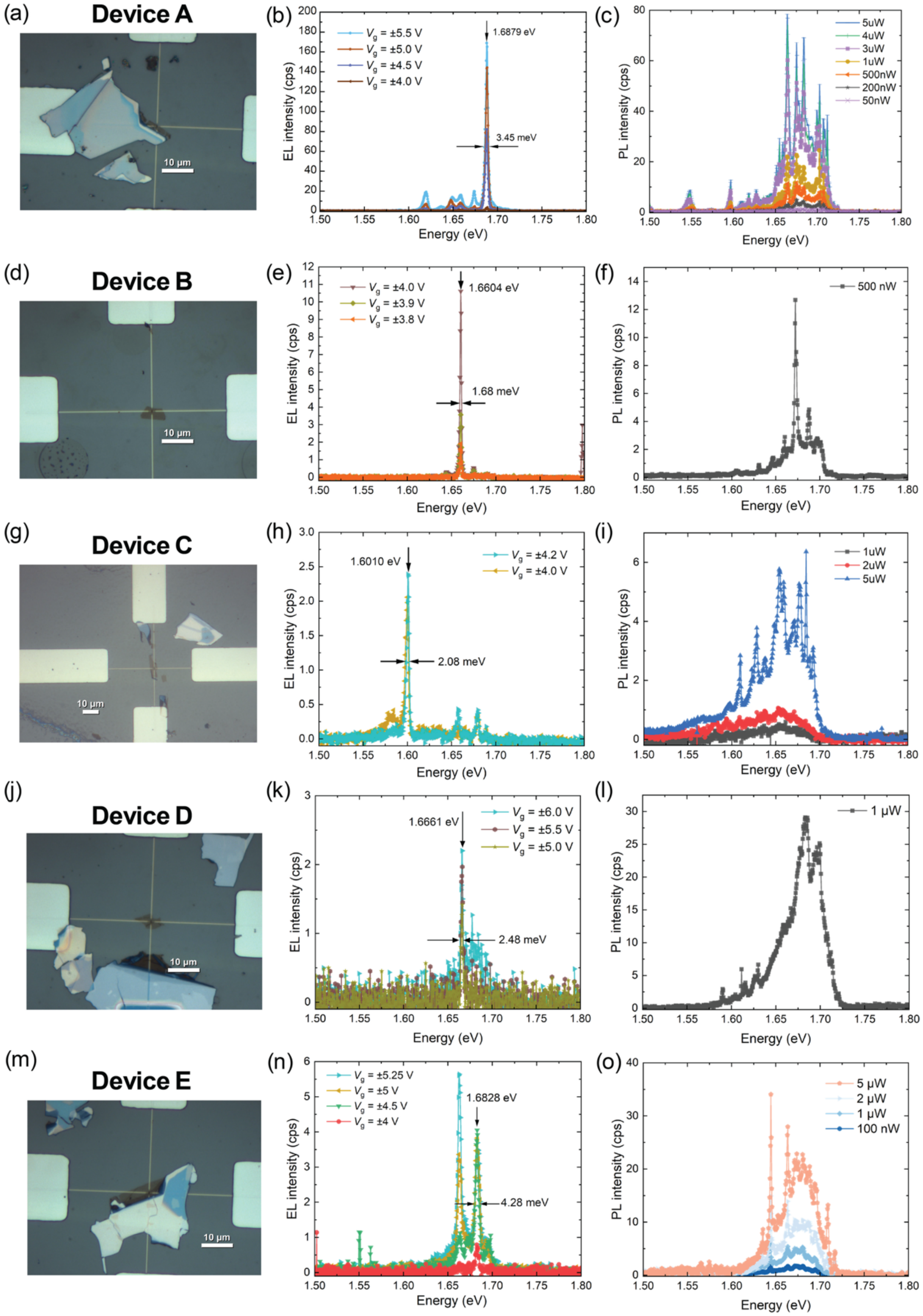

Figure S4. Device-to-device reproducibility of narrow-linewidth ACEL.

(a, d, g, j, m) Optical images of representative devices with monolayer WSe_2 positioned at the nanogap centers. (b, e, h, k, n) ACEL spectra collected from five different WSe_2 nanogap devices (Devices A–E) under 10 MHz AC excitation at various gate voltages. (c, f, i, l, o) Photoluminescence (PL) spectra of the same devices under continuous-wave 532 nm laser excitation, showing multiple emission peaks.

The PL spectra in Figures S4c, f, i, l, o show broad, multipeak emission even at low excitation power, with several features increasing together as the excitation power is raised. The ~1 μm diffraction-limited laser spot excites an ensemble of localized states within the illuminated region. In contrast, ACEL provides spatially confined electrical excitation within the sub-5 nm nanogap and preferentially addresses a dominant localized emitting state. This spatial selectivity suppresses ensemble averaging and produces a spectrally isolated, narrow-linewidth feature.

ACEL measurements were performed on 49 independent nanogap devices. Figure S4 presents spectra from five representative monolayer devices exhibiting sharp ACEL (Devices A – E; Figures S4a, d, g, j, m), all fabricated using the same HS-Au/Pt architecture and dry-transfer process.

Figures S4b, e, h, k, n show voltage-dependent ACEL spectra for these devices. The fitted Lorentzian peak energies and FWHM values for Devices A-E are 1.6879 eV and 3.45 meV, 1.6604 eV and 1.68 meV, 1.6010 eV and 2.08 meV, 1.6661 eV and 2.48 meV, and 1.6828 eV and 4.28 meV, respectively. Devices A-E in Figure S4 correspond to SA-03, SA-01, SA-04, SA-05, and SA-06 in Table S3, respectively. These results demonstrate narrow ACEL features with device-dependent emission energies and linewidths.

This variation may reflect differences in both the nanogap geometry and the local interfacial environment. Variations in the radius of spherical Au curvature and atomic-scale structure at the Au nanoparticle apex may modify the local strain and electronic environment in the WSe_2 monolayer. Variations in interfacial contact, including van der Waals adhesion, conformational wrinkling, and local dielectric screening, may also contribute to device-specific emission energies. The present data do not uniquely identify strain as the origin of the emitting localized states.

As the driving voltage increases, the primary ACEL peak saturates, and additional peaks emerge at both lower and higher energies. This sequential activation is consistent with preferential population of a dominant localized state within regions of enhanced electric field near the curved Au boundaries bordering the nanogap, followed by the activation of additional localized states as the carrier-injection level increases.

Table S2. Outcome distribution of the 49 measured devices.

Measured-device outcome	Count	Percentage of all devices
Sharp ACEL (monolayer)	14	28.6%
Sharp ACEL (bilayer)	1	2.0%
Broad/mixed ACEL	13	26.5%
No detectable ACEL	21	42.9%
**Total**	**49**	**100.0%**

The device-level analysis included 49 devices, all of which exhibited measurable current–voltage responses and detectable photoluminescence from WSe_2. The devices were classified according to their recorded electroluminescence spectra as monolayer sharp ACEL, bilayer sharp ACEL, broad/mixed ACEL, or no detectable ACEL. The corresponding outcome counts and fractions are summarized in Table S2. The bilayer device was retained in the overall outcome distribution but was not included in the statistical analysis of the monolayer devices.

Table S3. Device-level spectral and voltage data for 14 monolayer devices exhibiting sharp ACEL.

Device	Lowest recorded ACEL voltage, $\|V_g\|$ (V)	Voltage of fitted spectrum, $\|V_g\|$ (V)	Peak energy (eV)	Equivalent wavelength (nm)	Lorentzian FWHM (meV)
SA-01	3.60	4.00	1.6604	746.7	1.68
SA-02	4.60	5.20	1.6530	750.1	2.23
SA-03	4.00	5.50	1.6879	734.5	3.45
SA-04	4.00	4.20	1.6010	774.4	2.08
SA-05	5.00	5.50	1.6661	744.2	2.48
SA-06	4.00	4.50	1.6828	736.8	4.28
SA-07	5.50	7.00	1.6429	754.7	1.49
SA-08	4.00	4.20	1.6700	742.4	1.80
SA-09	4.00	4.50	1.6668	743.8	3.44
SA-10	7.50	7.50	1.6759	739.8	1.00
SA-11	7.80	8.10	1.6257	762.7	1.59
SA-12	6.75	6.75	1.7751	698.5	1.00
SA-13	4.80	4.80	1.6570	748.2	3.61
SA-14	6.50	6.50	1.6752	740.1	2.06

For each monolayer device exhibiting sharp ACEL, Table S3 reports the lowest gate-voltage amplitude at which an ACEL spectrum was recorded and the voltage of the spectrum selected for spectral fitting. Because measurements were performed at discrete voltage amplitudes and spectra at lower voltages were not systematically recorded, the lowest recorded ACEL voltage should not be interpreted as an exact turn-on threshold.

The selected emission feature was fitted using a Lorentzian main peak and a broader Gaussian component constrained to the red-shifted side. The peak energy and FWHM reported in Table S3 correspond to the Lorentzian component. The equivalent wavelength was calculated from the fitted peak energy using λ (nm) = 1239.84/E (eV). Devices showing only weak and comparatively broad spectral features were assigned to the broad/mixed ACEL category and were excluded from the monolayer sharp-line analysis. Across the 14 monolayer sharp-ACEL devices, the median fitted Lorentzian peak energy was 1.6664 eV (interquartile range: 1.6540–1.6757 eV), corresponding to a median wavelength of 744.0 nm (interquartile range: 739.9–749.6 nm). The median Lorentzian FWHM was 2.07 meV, with an interquartile range of 1.61–3.20 meV and a full range of 1.00–4.28 meV. The median lowest recorded ACEL voltage was 4.70 V, with an interquartile range of 4.00–6.25 V and a full range of 3.60–7.80 V.

Spatial ACEL profiles at elevated voltage amplitudes

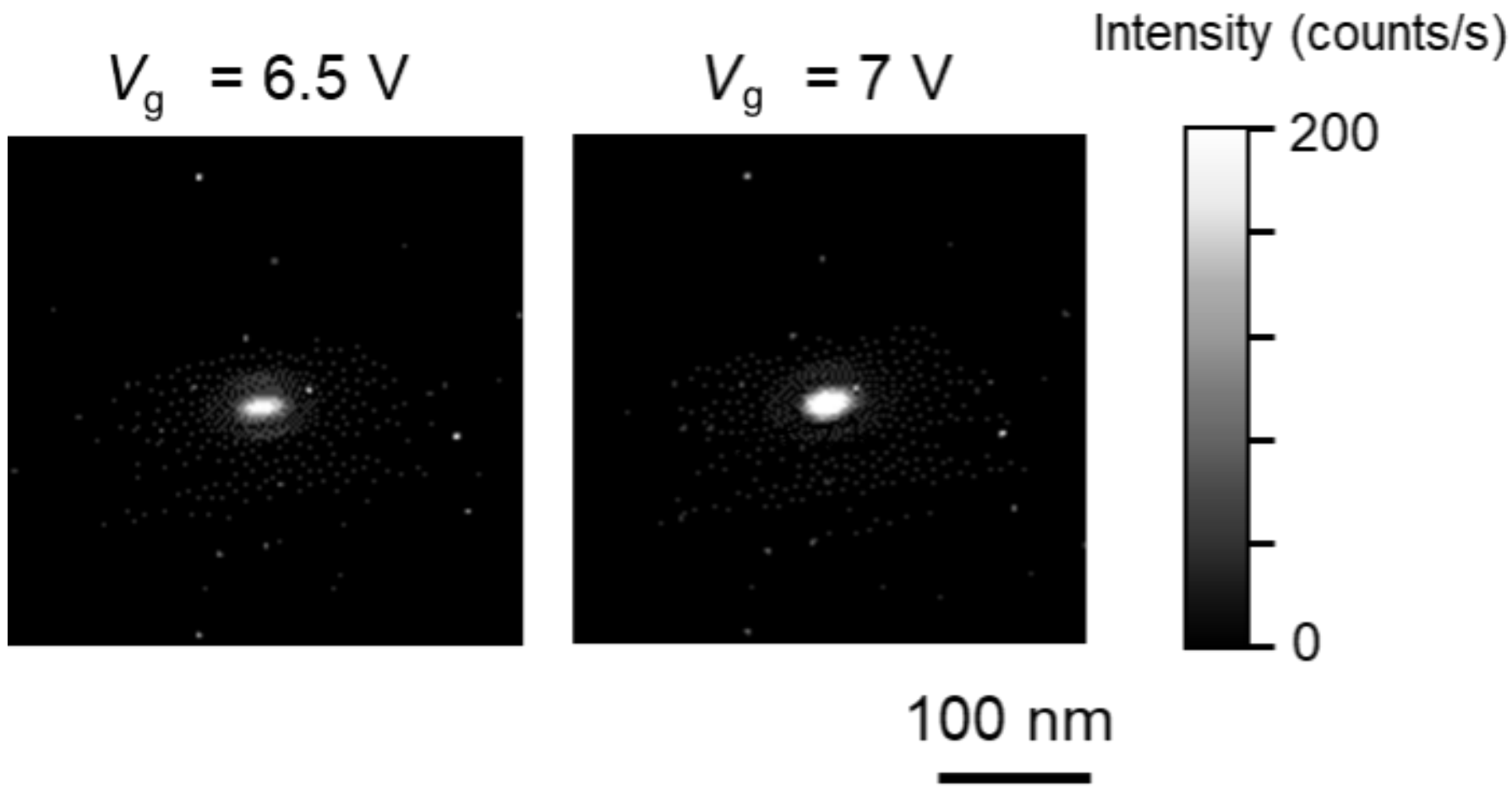

Figure S5. Evolution of the spatial ACEL profile at elevated driving voltages. Spatial emission maps acquired at $V_g = \pm 6.5$ and ± 7.0 V show that the initially localized emission develops an extended elliptical profile at higher voltage amplitudes. The elongation occurs predominantly along the nanowire direction, consistent with the population of additional neighboring emitting states. The anisotropic profile may reflect the local electrode geometry and strain distribution surrounding the nanogap.

This behavior is consistent with carrier spillover from the primary localized emitting state at the nanogap junction into neighboring states that become accessible at higher carrier injection levels. The anisotropic emission profile suggests that these additional emitting states are preferentially distributed along the nanowire direction, potentially reflecting the local device geometry and strain landscape surrounding the nanogap.

Schematic of conformal attachment and strain formation

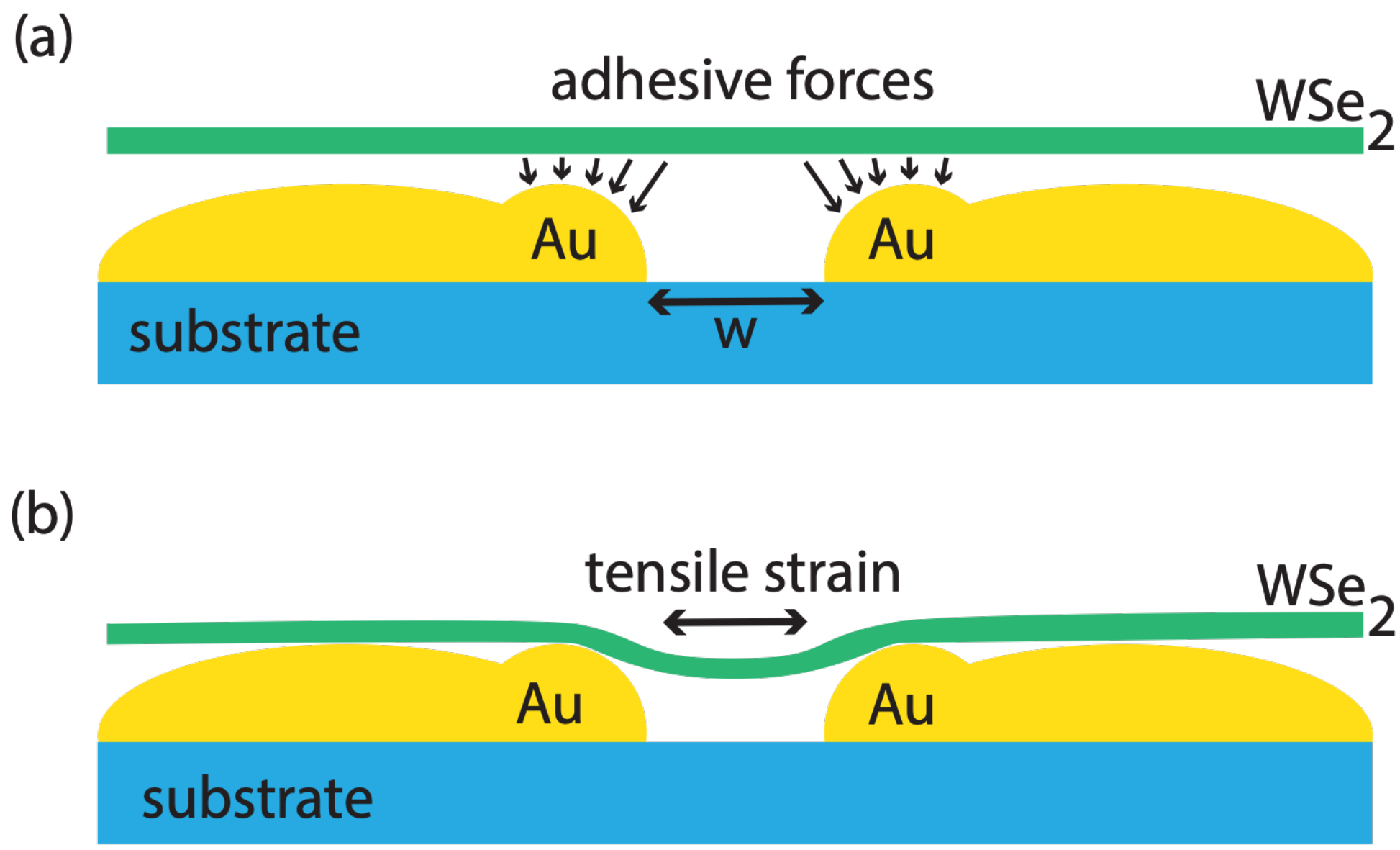

Figure S6. Conceptual illustration of adhesion-induced deformation of monolayer WSe_2 on curved Au contacts. The device width is defined as the separation between the Au nanoparticles. (a) As the WSe_2 monolayer approaches the Au contacts, interfacial adhesion promotes conformal attachment to the curved surfaces. The arrows schematically indicate the direction of the adhesion-induced deformation. (b) After relaxation, the monolayer bends around the Au contacts and bridges the intervening nanogap. This schematic illustrates the expected deformation but does not specify the sign, magnitude, or spatial distribution of the resulting strain. The nonuniform strain distribution is evaluated using the atomistic model presented in Figure S7.

Transfer of the WSe_2 monolayer onto the curved Au contacts is expected to produce interfacial adhesion and deformation of the monolayer. As illustrated conceptually in Figure S6, the monolayer can conform to the Au surfaces while bridging the approximately 4.5 nm separation measured in Figure 1c. This geometry may generate nanoscale strain near the junction. However, Figure S6 does not establish a uniformly tensile strain state or determine the magnitude and spatial distribution of the strain. These quantities depend on the contact geometry, interfacial interactions, transfer process, and thermal history. We therefore use the atomistic model described in Figure S7

to examine the types and approximate magnitudes of tensile and compressive strain that may arise from the curved Au-contact geometry.

Atomistic modeling of strain in the WSe_2 nanogap

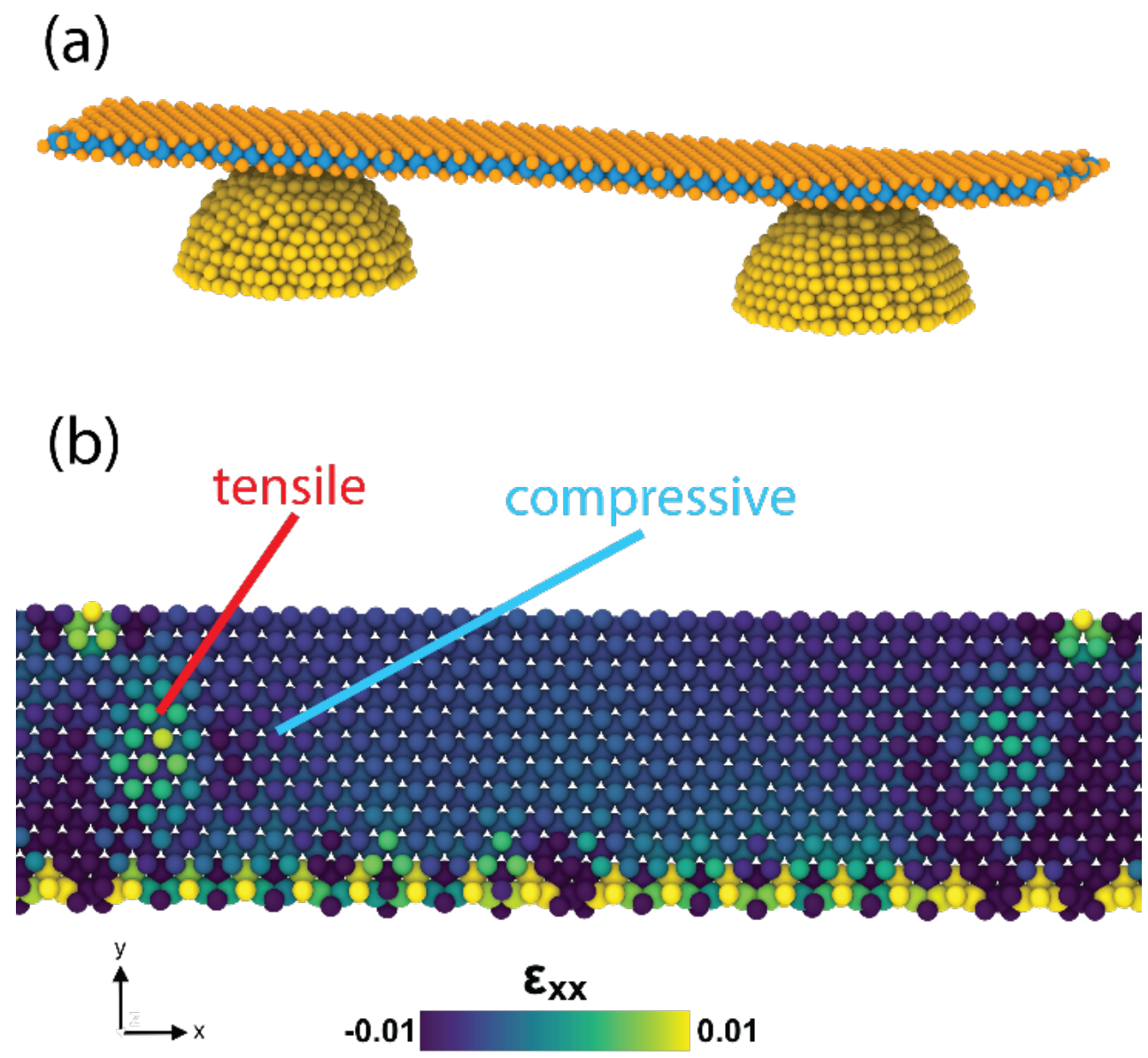

Figure S7. Atomistic modeling of strain in the WSe_2 nanogap. (a) The model used to explore the strain state is shown in its final, strained configuration. The gold atoms are Au, the blue atoms are W, and the orange atoms are Se. (b) ε_{xx} of the top of the film. The strains depicted at the edge of the film are not physical. The two broader, less brightly colored patches on the horizontal midline of the ribbon sit atop the Au hemispheres. These regions are stretched in tension because of the tendency for the film to wrap around the Au hemispheres, thus bending the film. Immediately adjacent to these regions are regions of compressive strain, again due to the bending configuration of the film. The local magnitudes of both tensile and compressive ε_{xx} approach 0.5%-1.0% and are rapidly varying with position.

The strain state in the WSe_2 monolayer suspended over the Au contacts is expected to depend on the assumed contact geometry, interfacial interactions, transfer process, and thermal history. To explore the types of nanoscale strain that may arise, we developed an atomistic model of a WSe_2 ribbon suspended between two Au hemispheres. The interactions between the atoms were modeled using a universal machine-learning interatomic potential (MLIP).[7] Universal MLIPs are generally less accurate than potentials trained for a specific material system. Accordingly, the calculated strain distribution is intended as a qualitative estimate of the types and approximate

magnitudes of strain that may arise, rather than as an exact reconstruction of the experimental strain field.

The geometry of the studied system is presented in Figure S7a. The Au hemispheres are shown as gold atoms, W atoms are blue, and Se atoms are orange. The model reproduces the opposing hemispherical-contact topology but is not a quantitatively scaled reconstruction of the experimentally measured 4.5 nm gap. In this image, the centers of the hemisphere bases are separated by 101 Å. This configuration of atoms was created by first minimizing the energy of a hemispherical Au island, copying it and rotating it by 180-degrees about an axis normal to the base plane of the hemisphere, and arranging hemispheres so that they are separated by 100 Å. A WSe_2 ribbon was draped across the islands, and the total energy was minimized using ASE.[8] During the minimization (and all subsequent steps), the Au atoms in the plane of the hemispheres were fixed in position. Then, a molecular dynamics simulation (also using ASE within an NVE ensemble) was run for 10,000 1 fs time steps with a temperature set near 300 K. The final frame of the MD simulation was then used as the starting point for an energy minimization step. Finally, the distance between the centers of the Au hemispheres was increased by 0.02 Å, and the film and unfixed Au atoms relaxed. This was repeated 50 times until the separation increased to 101 Å. The systems were relaxed using the FIRE algorithm until the maximum residual force on any of the atoms was reduced to less than 0.01 eV/ Å. The atomic strain was then computed using OVITO.[9]

Figure S7b shows a top view of the resulting strain (ε_{xx}) distribution in the WSe_2 ribbon at the end of the calculation. Note that the strains reported at the edge of the ribbon are artifacts - the strain is not well defined at the edges. The two relatively large lighter regions at the ends of the mid-line of the ribbon sit immediately atop the Au hemispheres. These regions display a tensile strain, largely because the film slightly wraps around the hemispheres and the associated bending stretches this part of the film. Immediately adjacent to these tensile regions are regions of compressive strain. These arise because the film is stretching towards the adjacent island. Both of these strains are approaching 0.5%-1.0%. The net effect is that the film displays a highly non-uniform strain state after processing.

Numerical electrostatic simulations.

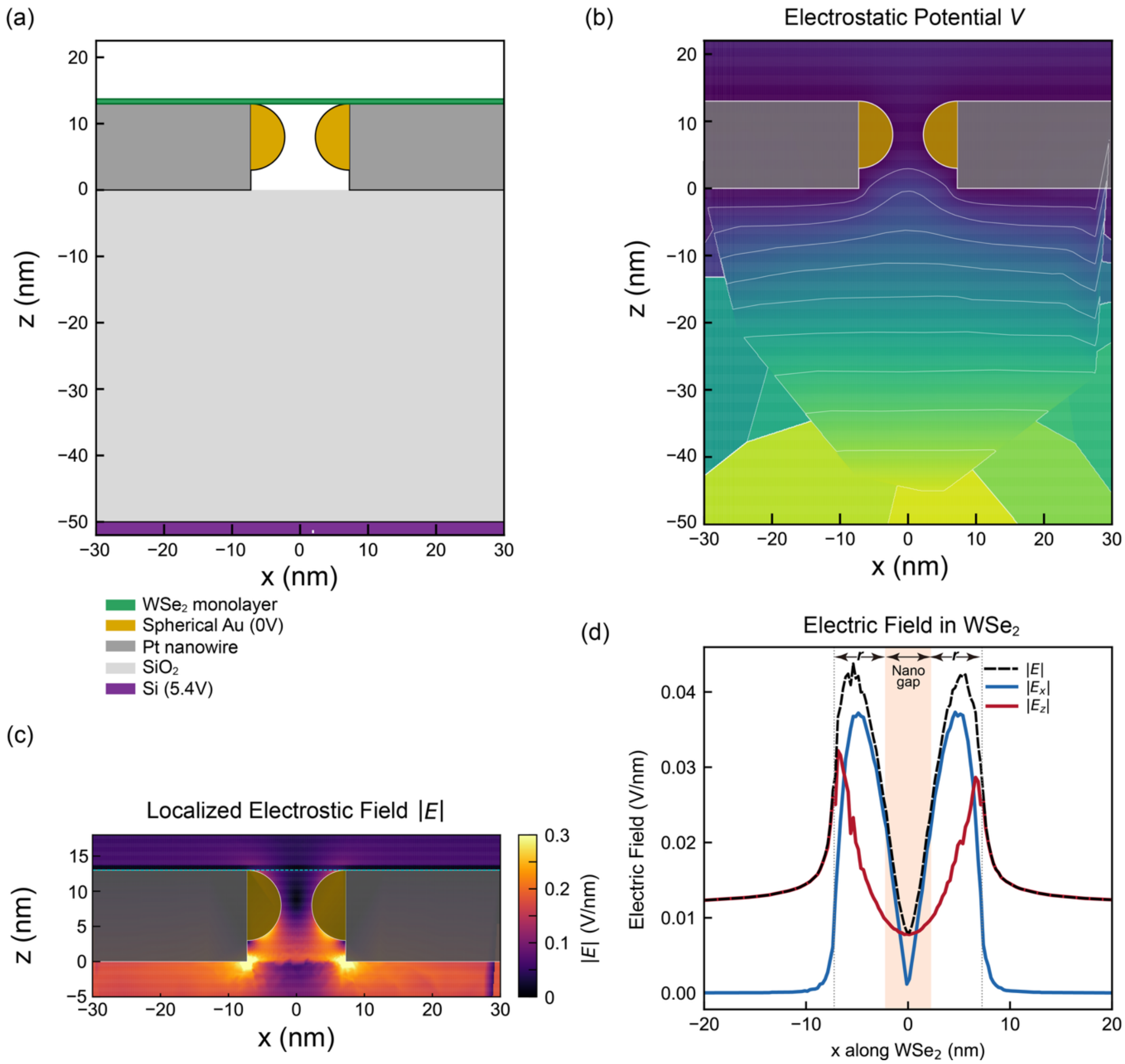

Figure S8. Numerical electrostatic simulation of the WSe_2 nanogap device.

(a) Cross-sectional geometry and electrical boundary conditions used in the three-dimensional finite-element simulation. Two grounded Pt/Au electrodes with spherical Au tips (radius: 5 nm) are separated by a 4.5 nm gap on a 50 nm-thick SiO_2/Si substrate, and monolayer WSe_2 covers the electrodes and nanogap. The Pt nanowires are 15 nm wide and 13 nm high. The Si back gate is biased at V_g = 5.4 V, while both Pt/Au electrodes are held at 0 V. (b) Electrostatic-potential distribution in the central x-z plane. (c) Corresponding electric-field magnitude, |E|, near the nanogap. The cyan dashed line indicates the WSe_2 plane. (d) Spatial profiles of the total electric-field magnitude, |E|, and its in-plane and out-of-plane components, $|E_x|$ and $|E_z|$, respectively,

evaluated along WSe_2. The shaded region indicates the 4.5 nm nanogap. The electric field is strongly localized near the curved Au electrode boundaries, whereas the in-plane component approaches zero at the gap center because of the symmetric electrode configuration.

The electrostatic-potential distribution was calculated by solving the three-dimensional Laplace equation,

$$\nabla \cdot (\varepsilon_r \nabla V) = 0,$$

using the finite-element method. The relative dielectric constants of SiO_2 and WSe_2 were set to 3.9 and 5, respectively, and the surrounding medium was treated as vacuum with a relative dielectric constant of 1. The Pt and Au regions were treated as ideal equipotential conductors and fixed at 0 V. The bottom Si boundary was fixed at $V_g = 5.4$ V, and electrically insulating boundary conditions were applied to the outer boundaries of the simulation domain. The electric field was calculated from the simulated potential using $\mathbf{E} = -\nabla V$.

A locally refined tetrahedral mesh was used to resolve the curved Au tips, the 4.5 nm gap, and the monolayer WSe_2 region. Three mesh resolutions with minimum local element sizes of 0.50, 0.35, and 0.25 nm were examined. The change in the mean electric-field magnitude in WSe_2 between the 0.35 and 0.25 nm meshes was 0.89 percent, confirming that the calculated field distribution was sufficiently converged. The results shown in Figure S8 were obtained using the finest mesh, with a minimum local element size of 0.25 nm.

As shown in Figures S8b and S8c, the applied back-gate voltage produces a strongly nonuniform potential and electric-field distribution near the nanogap. The electric field is concentrated near the curved Au electrode boundaries adjacent to the gap. Along WSe_2, the in-plane field component, $|E_x|$, reaches approximately 0.037 V/nm near the Au/WSe_2 boundaries, and the corresponding total electric-field magnitude, $|E|$, reaches approximately 0.044 V/nm. The out-of-plane component, $|E_z|$, is also enhanced near these boundaries because of capacitive coupling between the back gate and the grounded electrodes.

At the exact center of the nanogap, $|E_x|$ approaches zero because the lateral electric-field contributions from the two symmetrically grounded electrodes cancel. Therefore, the simulation

indicates that the regions of enhanced electric field are spatially confined near the two Au/WSe_2 contact boundaries bordering the nanogap rather than exactly at its geometrical center.

The present calculation is a quasistatic electrostatic analysis and does not explicitly include transient current, carrier transport, trap occupancy, or microscopic trapping and detrapping kinetics. The simulation is therefore used to quantify the gate-induced potential and electric-field distributions and their spatial localization, rather than to reproduce the complete time-dependent carrier-recombination process.[5,6]

ACEL response under sinusoidal excitation

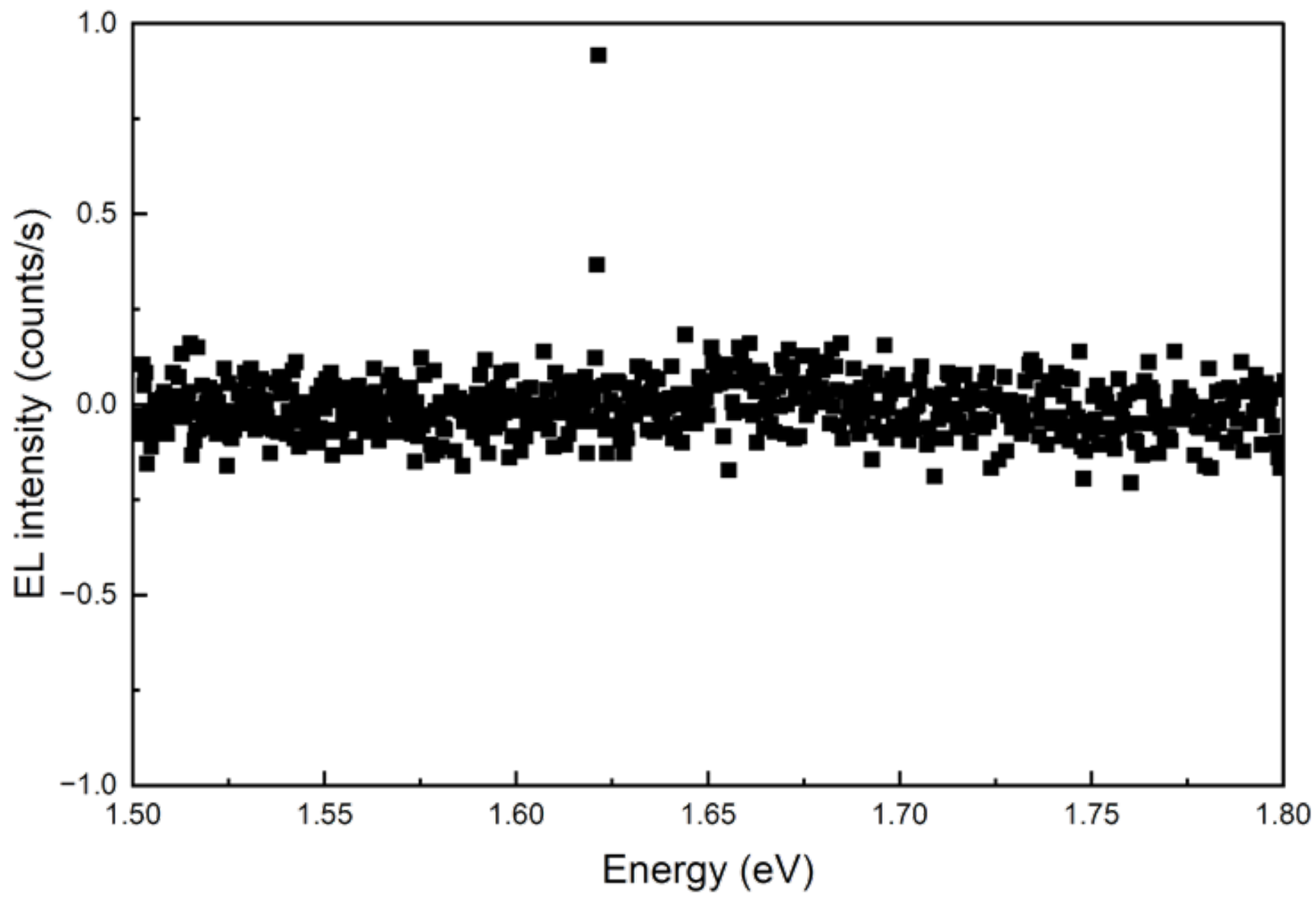

Figure S9. ACEL under sinusoidal excitation (10 MHz, V_g = ±4.2 V).

The rapid voltage transitions of the square-wave excitation produce a larger voltage slew rate and are therefore expected to generate a stronger capacitive displacement-current transient than sinusoidal excitation.[5,6] The square wave also maintains the applied field near its maximum magnitude for a larger fraction of each half-cycle. These differences are consistent with more efficient transient carrier injection under square-wave excitation. Because the transient current was not measured directly, this interpretation does not constitute a quantitative determination of the injection-current waveform.

Frequency-dependent ACEL spectra from 1 to 3 MHz

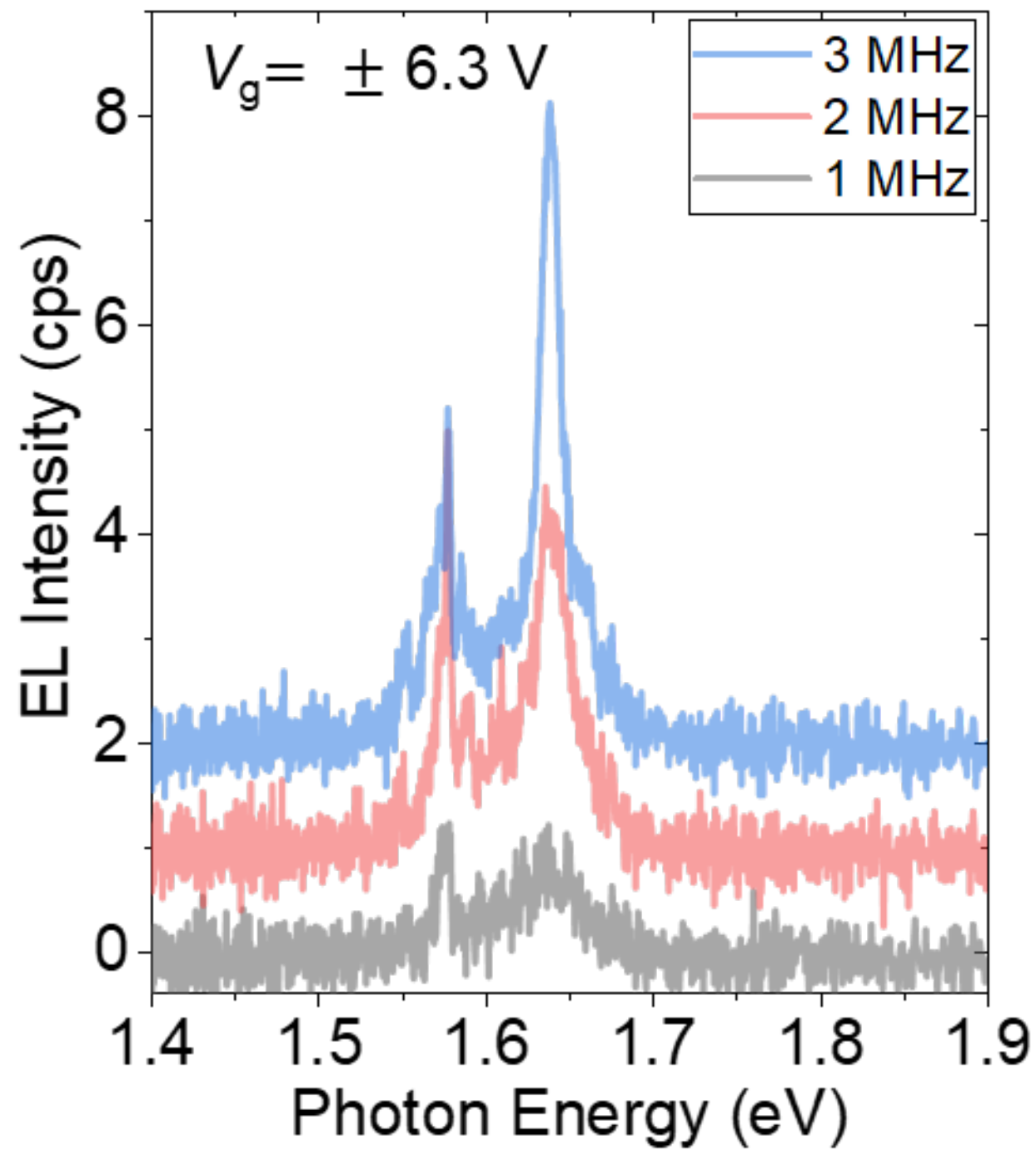

Figure S10. Low frequency-dependent ACEL spectra acquired from an independent nanogap device under square-wave excitation at 1, 2, and 3 MHz.
Successive spectra are vertically offset by 1 cps for clarity. The apparent FWHM values of the composite emission envelope near 1.64 eV are approximately 47, 24, and 14 meV at 1, 2, and 3 MHz, respectively.

Figure S10 presents ACEL spectra acquired from the same independent nanogap device under square-wave excitation at 1, 2, and 3 MHz. The voltage amplitude, integration time, optical configuration, and data-processing procedure were kept unchanged across the three measurements. The spectra are displayed with successive vertical offsets of 1 cps for clarity.

The emission feature near 1.64 eV increases in intensity and becomes better resolved with increasing frequency. The background-referenced peak height is approximately 1 cps at 1 MHz, 3 cps at 2 MHz and 6 cps at 3 MHz. Meanwhile, the apparent FWHM of the composite emission envelope decreases from approximately 47 meV at 1 MHz to 24 meV at 2 MHz and 14 meV at 3 MHz. The spectra contain several partially overlapping features superimposed on a broad

residual background. The reported widths characterize the overall emission envelope rather than the intrinsic linewidth of an individual emission line. In addition, the lower signal-to-background ratio at the lower frequencies may contribute to the larger apparent envelope width.

The half-cycle durations of the applied square wave are 500, 250, and 167 ns at 1, 2, and 3 MHz, respectively. During the longer half-cycles at lower frequencies, carriers may undergo redistribution, trapping, and detrapping through a broader range of localized states, resulting in multiple overlapping emission features and a broad spectral background. Increasing the frequency increases the number of field-reversal and transient injection events per unit time, which can contribute to the higher time-averaged ACEL intensity. The accompanying reduction in the half-cycle duration may also limit slower redistribution pathways and favor repeated population of a smaller subset of more rapidly responding or more strongly coupled localized states, consistent with the observed narrowing of the composite emission envelope. Because the carrier and trap dynamics were not measured directly, and because the signal-to-background ratio changes with frequency, this explanation should be regarded as a possible interpretation rather than a determination of microscopic trapping, detrapping, or relaxation times.

Because the spectra at 1–3 MHz and the 10 MHz spectrum presented elsewhere were acquired from different devices, they are not used to construct a continuous frequency-response curve or determine an optimal operating frequency. In addition, the available waveform generator source was limited to a maximum frequency of 10 MHz under the voltage conditions required for the ACEL measurements.

Voltage-transition-time-dependent ACEL spectra

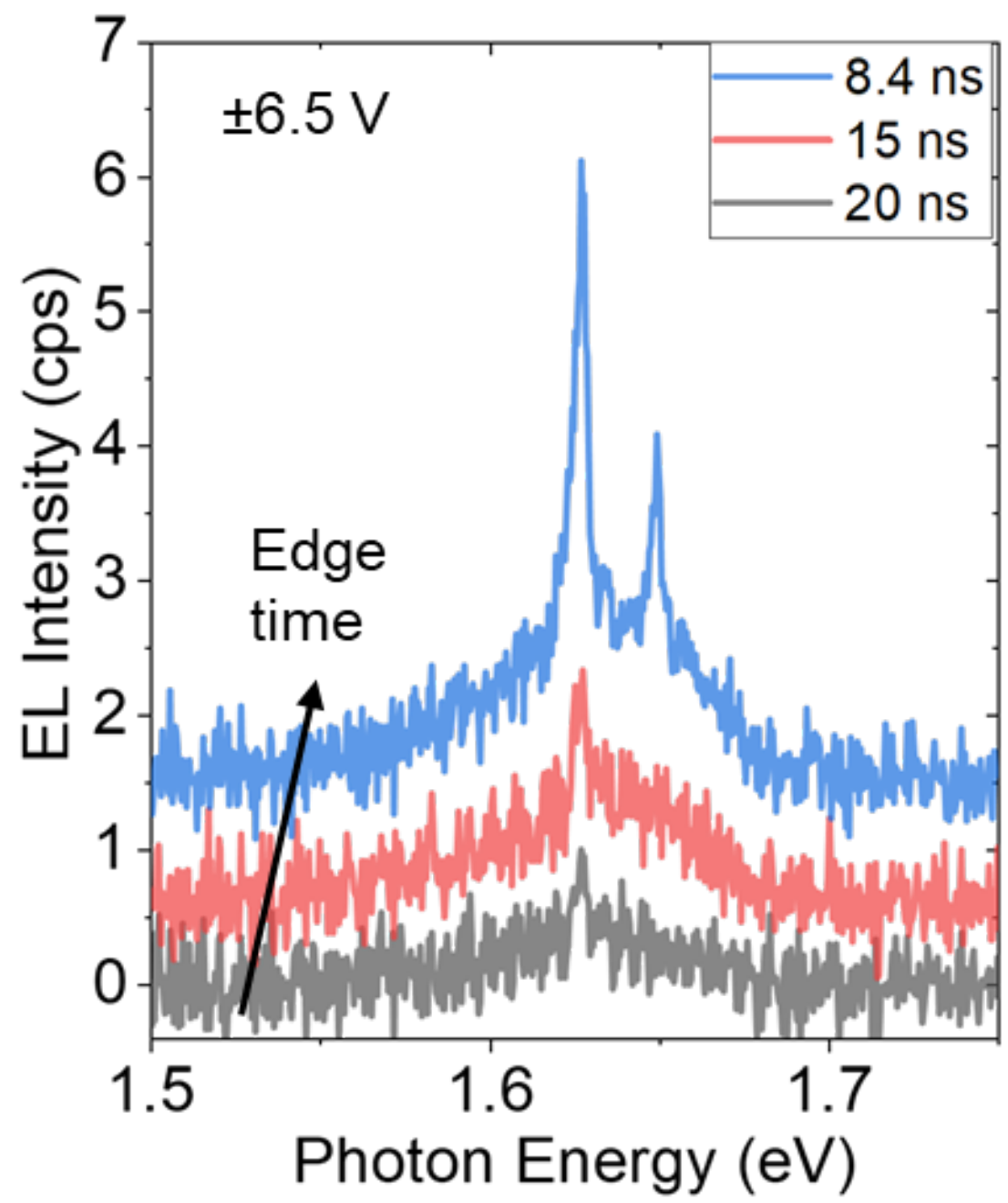

Figure S11. **Slew-time-dependent ACEL spectra acquired from an independent nanogap device at voltage-transition times of 20, 15, and 8.4 ns.**

The 20 ns spectrum is shown without an offset, whereas the 15 and 8.4 ns spectra are vertically offset by 0.5 and 1.5 cps, respectively, for clarity. A transition time of 8.4 ns was the minimum achievable using the waveform generator employed in this experiment. Shortening the transition time produces a more pronounced and spectrally better-resolved localized emission feature.

The influence of the voltage-transition time was examined in an additional nanogap device using square-wave excitation with slew times of 8.4, 15, and 20 ns, while the voltage amplitude, excitation frequency, and acquisition conditions were kept unchanged (Figure S11). This device was distinct from that used for the frequency-dependent measurements in Figure S10. As the slew time was shortened from 20 to 8.4 ns, the localized ACEL feature became progressively more pronounced and spectrally better resolved. A shorter transition time corresponds to a larger $|dV_g/dt|$ and is expected to produce a stronger capacitive displacement-current transient and more rapid modulation of the nanogap potential. The observed spectral evolution therefore supports

the importance of abrupt voltage transitions in promoting transient carrier injection and localized radiative recombination.

Optical and electrical characterization of 2 μm control devices

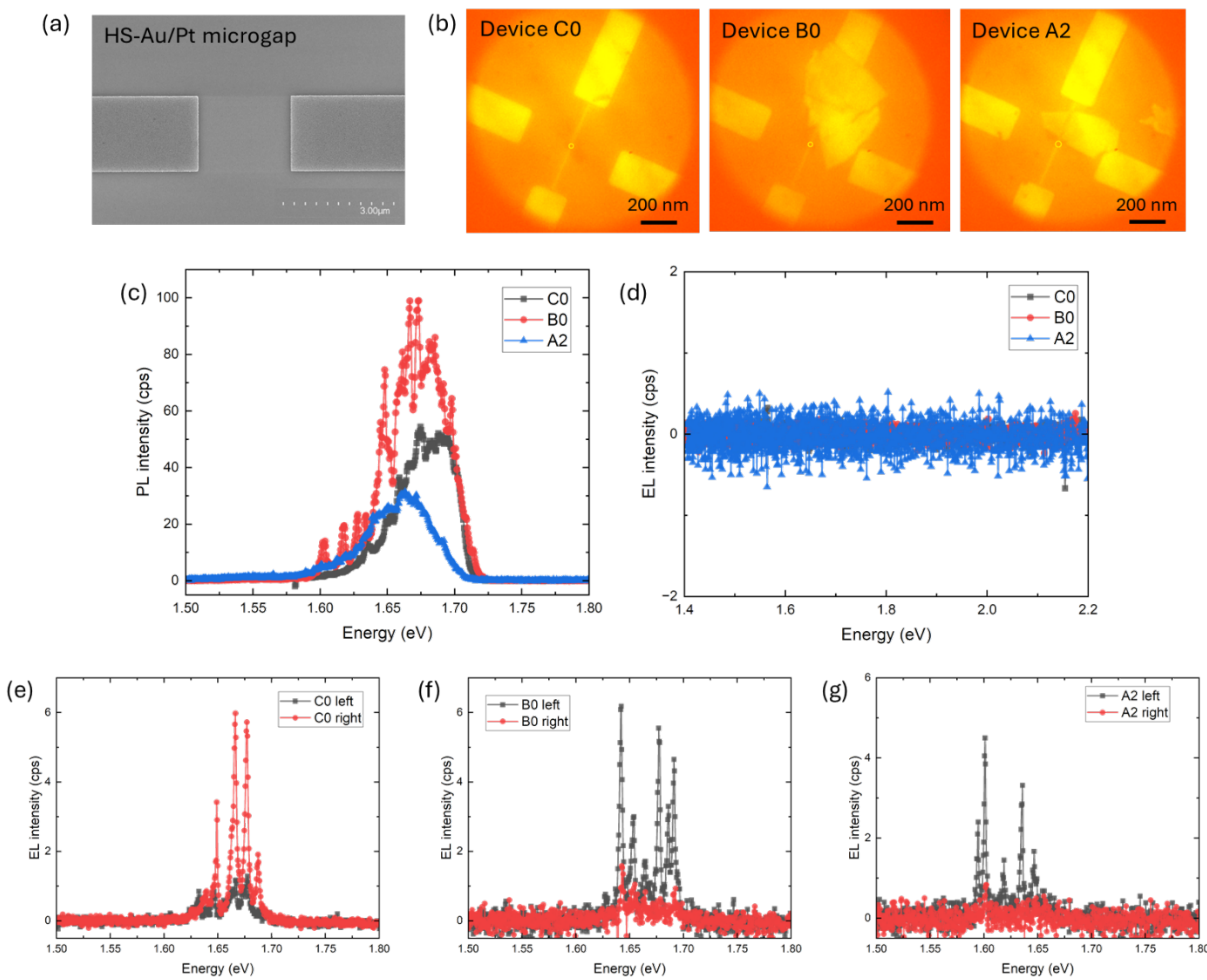

Figure S12. Optical and electrical characterization of 2 μm control devices.

(a) SEM image of a 2 μm gap prior to monolayer transfer. (b) Optical images of three control devices with a 2 μm channel covered by monolayer WSe_2 (50× objective, 550 nm filter). The yellow circle indicates the laser spot. (c) PL spectra from three devices (Devices C0, B0, and A2; 532 nm CW laser, 500 nW, 180 s integration). (d) Corresponding ACEL spectra of the 2 μm channels, showing no detectable ACEL at the channel center even at $V_g = \pm 8.5$ V. (e–g) ACEL spectra measured at the electrode edges for (e) Device C0, (f) Device B0, and (g) Device A2, with the detection spot positioned on the left (black) or right (red) edge ($V_g = \pm 8.5$ V).

The 2 μm gap devices were fabricated following the same process described in the Methods and the "HS-Au/Pt nanogap fabrication and structural characterization" section of the Supporting Information, including electroless gold plating (ELGP) to form Au nanoparticles on the Pt electrodes (10 nm Pt/3 nm Ti).

PL from these devices shows a broad emission envelope centered at 1.66–1.68 eV without resolved narrow features, indicating that the widely spaced electrodes do not provide the spatial selectivity observed for the sub-5 nm nanogap devices.

Under AC excitation with both nanogap electrodes grounded, no electroluminescence is detected from the channel center. When the collection spot is shifted to the WSe_2/electrode interface, where the electrode edge may modify the local structural and electronic environment, a weak ACEL signal (<10 cps) is observed with one electrode grounded. However, the emission consists of multiple closely spaced peaks over a broad energy range, consistent with the behavior observed in the 35 nm side-channel devices.

This contrast with the sub-5 nm nanogap devices highlights the importance of nanoscale geometric and electric-field confinement. Although the 2 μm electrode edges may introduce local structural and field gradients, the resulting perturbation is distributed over a much larger spatial region than in the sub-5 nm nanogap devices. Consequently, no dominant localized emitting state is observed, and the measured ACEL consists of multiple emission features with substantially reduced intensity.

These control experiments indicate that sub-5 nm geometric and electric-field confinement is important for the emergence of spectrally isolated narrow-linewidth ACEL, while the role of strain in determining the emitting state cannot be uniquely established.

Effect of a 1.5 nm SiO_x interfacial spacer

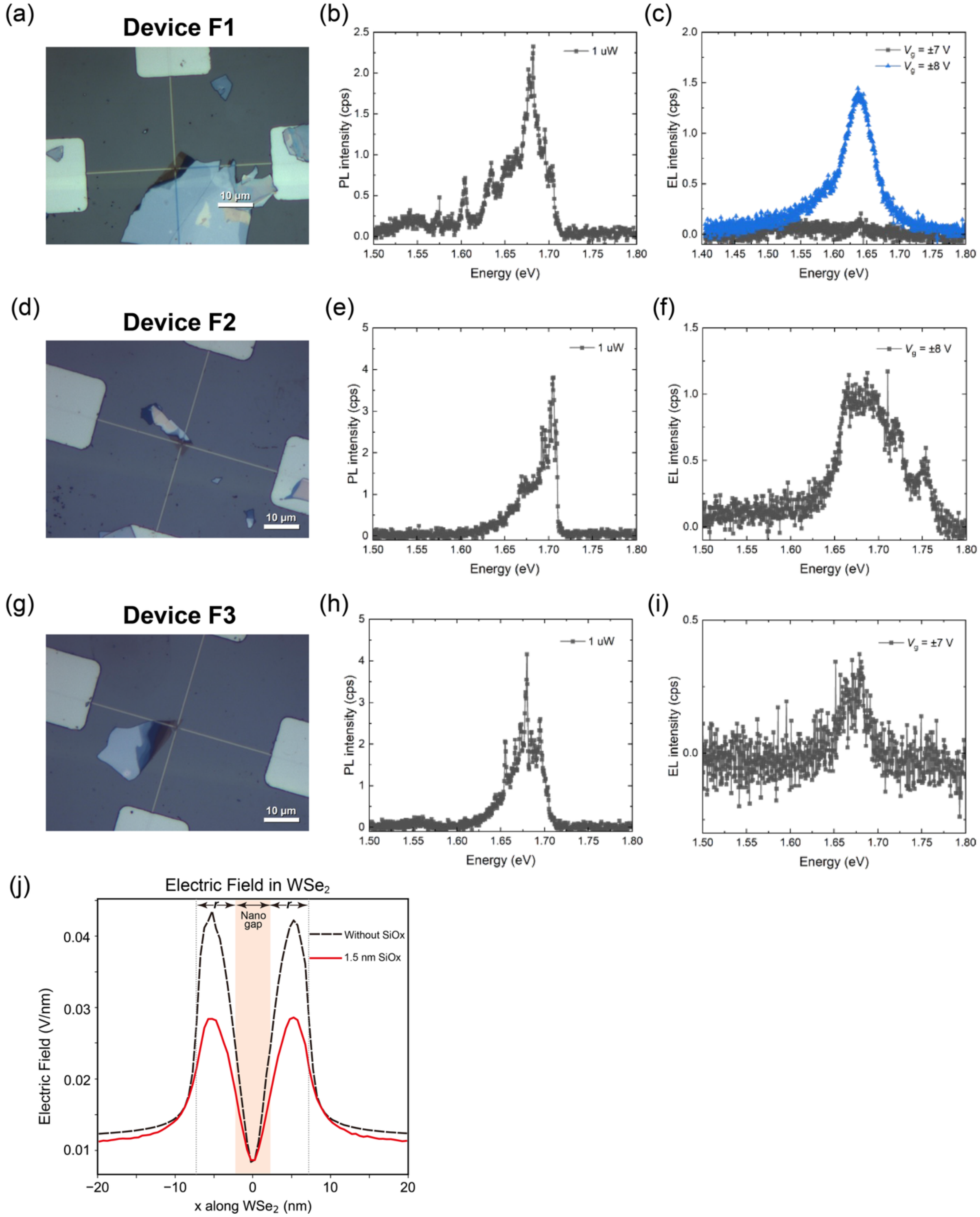

Figure S13. Impact of a 1.5 nm SiO_x interfacial spacer on ACEL spectra.

(a, d, g) Optical images of devices F1–F3. (b, e, h) PL spectra of the three control devices containing a 1.5 nm thermally evaporated SiO_x spacer between the electrodes and the monolayer

WSe_2 (532 nm excitation). (c, f, i) Corresponding ACEL spectra showing weak and broad emission under 10 MHz excitation. (j) Calculated electric-field magnitude, |E|, along WSe_2 for devices without SiO_x and with a 1.5 nm thermally evaporated SiO_x layer on the exposed Pt/Au electrode surfaces. The shaded region indicates the 4.5 nm metal-to-metal nanogap. The dotted vertical lines and arrows indicate the centers and radii (r = 5 nm) of the opposing curved Au tips, respectively.

Additional control devices were fabricated by inserting a 1.5 nm SiO_x dielectric spacer, deposited by thermal evaporation, between the electrodes and the subsequently transferred monolayer WSe_2. The PL spectra of three devices (Figure S13) show multiple emission peaks, indicating the presence of multiple localized emitting states at different energies. This suggests that the thin oxide layer does not eliminate spatial variations in the local structural and electronic environment of WSe_2; however, the microscopic origin of these states is not uniquely determined.

However, in contrast to devices without the SiO_x spacer, no sharp ACEL peak is observed. Instead, only broad emission appears at relatively high V_g, suggesting less selective carrier injection across the dielectric interface.

To evaluate the electrical effect of the thermally evaporated spacer, three-dimensional finite-element electrostatic simulations were performed using identical Pt/Au nanogap geometries for devices without and with SiO_x. In the SiO_x-containing model, a 1.5 nm-thick SiO_x layer with a relative dielectric constant of 3.9 covers the exposed Pt and Au electrode surfaces rather than forming a continuous planar layer beneath WSe_2. The metal-to-metal gap is 4.5 nm, and the minimum separation between the opposing SiO_x surfaces is 1.5 nm. The Pt/Au electrodes were fixed at 0 V, the Si back gate was biased at V_g = 5.4 V, and the surrounding medium was treated as vacuum. The calculated electric field in WSe_2 remains strongly localized near the curved Au electrode boundaries. However, the peak field along the plotted WSe_2 profile decreases from approximately 0.044 V/nm without SiO_x to approximately 0.029 V/nm with the 1.5 nm SiO_x layer, corresponding to a reduction of approximately 34%. This result indicates that the SiO_x layer reduces the localized electrode-edge field and weakens the direct electrical coupling

between the Pt/Au electrodes and WSe_2. The reduced coupling is consistent with the weak and broad ACEL observed experimentally in the SiO_x-spacer control devices.

This contrast between PL and ACEL highlights the distinct structural and electrical roles of the nanogap electrodes. The spectral broadening observed in ACEL is therefore unlikely to result simply from the absence of localized emitting states. Instead, the insertion of the dielectric spacer modifies the carrier-injection pathway and reduces the direct coupling between the electrodes and the WSe_2 monolayer. The resulting broad emission suggests that carrier injection becomes less selective, preventing selective population of a dominant subset of localized states.

These results indicate that a direct electrode/WSe_2 interface plays an important role in enabling selective electrical access to localized states and the emergence of narrow-linewidth ACEL.

Note 1. Spectral decomposition and estimation of the effective Huang–Rhys factor

The dark-subtracted ACEL spectrum acquired at 3.5 K was analyzed in OriginPro over 1.645–1.660 eV using an empirical additive two-component model. The narrow main feature was represented by a Lorentzian component, $I_L(E)$, and the red-shifted contribution was represented by a Gaussian component, $I_G(E)$. Their center energies were fitted independently, with $E_G < E_L$. The resulting function is an additive Lorentzian-plus-Gaussian model:

$$I(E) = I_L(E) + I_G(E)$$

$$I_L(E) = \frac{A_L(\frac{w_L}{2})^2}{(E - E_L)^2 + (\frac{w_L}{2})^2}$$

$$I_G(E) = A_G \exp[-\frac{(E - E_G)^2}{2\sigma^2}]$$

This additive model is distinct from a Voigt function, in which the Lorentzian and Gaussian broadening contributions are convoluted about a common center. Here, the Lorentzian and Gaussian components have independent center energies and are summed rather than convolved. Here, A_L and A_G are the fitted peak amplitudes, w_L is the Lorentzian FWHM, and σ is the Gaussian standard deviation. The Lorentzian and Gaussian components were assigned phenomenologically to the dominant zero-phonon line (ZPL) emission and a broader phonon sideband (PSB) contribution. No instrumental-response deconvolution was applied.

The component intensities were evaluated from the analytical integrals of the complete fitted functions:

$$I_{ZPL} = \pi\frac{A_L w_L}{2}, \qquad I_{PSB} = A_G\sigma\sqrt{2\pi}$$

At 3.5 K, the fit yielded E_L = 1.652717 eV, w_L = 1.9725 meV, A_L = 9.650 cps, E_G = 1.651720 eV, σ = 1.619 meV, and A_G = 5.907 cps. These parameters give I_{ZPL} = 0.02990 cps·eV and I_{PSB} = 0.02397 cps·eV, corresponding to an integrated-area ratio I_{ZPL}/I_{PSB} = 1.25.

Under the Debye–Waller approximation, the zero-phonon fraction is related to the Huang–Rhys factor, S, by

$$DW = \frac{I_{ZPL}}{I_{ZPL} + I_{PSB}} = e^{-S}, \quad S = -\ln(DW)$$

The fitted area ratio gives $DW = 0.555$ and an effective Huang–Rhys factor of $S = 0.589$ (approximately 0.59). This value suggests relatively weak electron–phonon coupling within the adopted spectral decomposition. Because this value depends on the phenomenological component assignment and does not account for the instrumental response, it should be regarded as an effective, model-dependent parameter rather than an intrinsic material constant.

Extended HS-Au/Pt nanowire control device

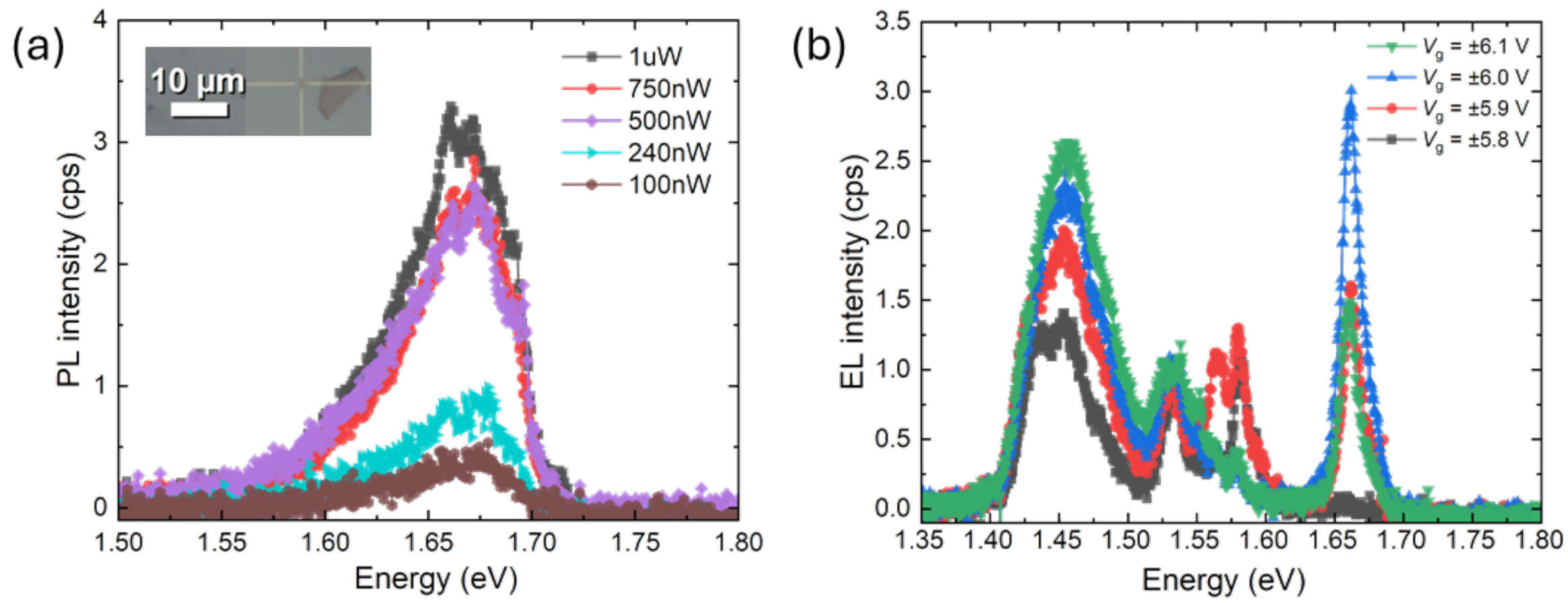

Figure S14. Optical and ACEL characteristics of a multi-defect one-dimensional (1D) nanowire device. (a) Power-dependent PL spectra of a monolayer WSe_2 transferred onto a continuous HS-Au/Pt nanowire (inset: optical image of the device). (b) Voltage-dependent ACEL spectra measured under 10 MHz excitation.

A monolayer WSe_2 was transferred onto a continuous HS-Au/Pt nanowire. Optical emission measurements were performed under AC excitation with the right electrode grounded as the source and the left electrode floating.

Under optical excitation, the PL spectrum exhibits a broad emission envelope with weak defect-related features that do not resolve into narrow lines, consistent with emission from multiple localized states distributed along the nanowire rather than a dominant emitting region.

Under AC excitation, the ACEL spectrum shows multiple emission lines at distinct energies (~1.45, ~1.55, and ~1.66 eV), each with a linewidth exceeding 5 meV, more than twice that of the primary nanogap device. The broad energy range (1.45–1.66 eV) suggests contributions from multiple localized states with different confinement energies.

As in the nanogap devices, the emission intensity increases and additional peaks emerge with increasing V_g, reflecting a field-driven injection process. However, the multi-peak spectrum with broad linewidths indicates that carriers are distributed among competing trap states along the nanowire axis, rather than being funneled into a dominant subset of localized states.

These results indicate that neither local structural variation nor electric-field enhancement alone is sufficient to produce spectrally isolated narrow-linewidth ACEL. The sub-5 nm nanogap geometry spatially confines regions of enhanced electric field and may also modify the local strain landscape, thereby favoring selective electrical access to localized states. The present controls do not uniquely establish strain as the microscopic origin of the emitting state.

Bilayer WSe_2 nanogap device

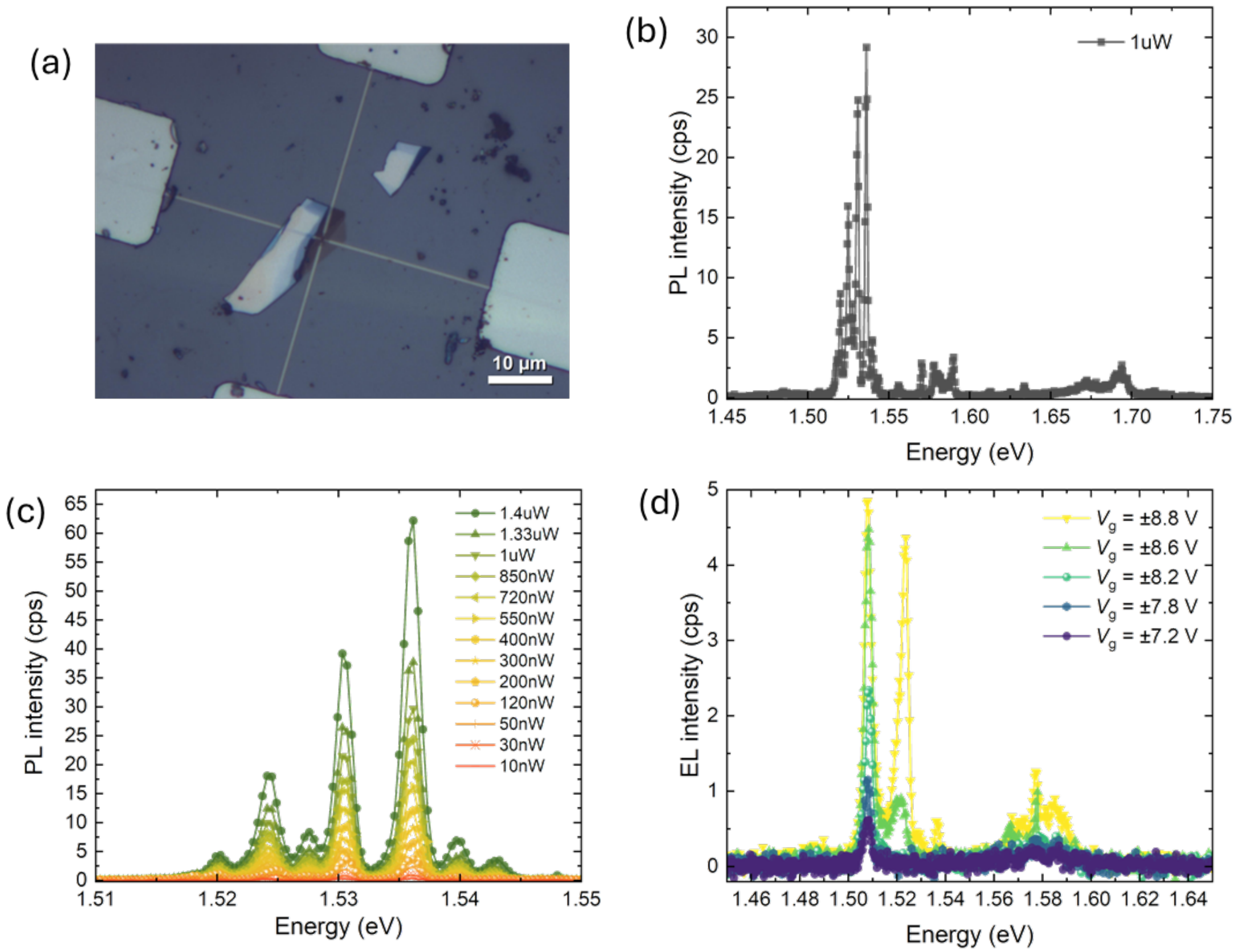

Figure S15. Optical and ACEL characteristics of a bilayer WSe_2 nanogap device. (a) Optical image of the sub-5 nm nanogap device where a bilayer WSe_2 flake is at the center. (b) PL spectrum of the bilayer WSe_2 device measured at 3.5 K. (c) Power-dependent PL from 1.51 eV to 1.55 eV. All PL spectra were acquired under 532 nm excitation. (d) Voltage-dependent ACEL spectra acquired under 10 MHz excitation.

The PL spectrum is dominated by intense narrow peaks in the 1.52–1.54 eV range. Such emission may be influenced by the local structural environment of bilayer WSe_2 at 3.5 K, because nanoscale strain can partially lift the indirect-bandgap character of the bilayer.[10] Additional localized-state-related peaks appear at 1.56–1.58 eV, together with broader emission features at 1.65–1.70 eV.

Under AC excitation, a dominant narrow emission line emerges at ~1.506 eV above V_g = 7.2 V. This peak lies ~20 meV below the PL band edge and is consistent with emission from a localized state in the bilayer, although its microscopic origin is not uniquely determined. As the voltage increases, the primary peak saturates, and secondary peaks appear at 1.52 eV and 1.58 eV, following the same sequential activation behavior observed in monolayer devices.

These results suggest that the sub-5 nm nanogap architecture is not limited to monolayer WSe_2 and can support narrow-linewidth ACEL from localized states in bilayer systems as well.

Monolayer MoS_2 nanogap control devices

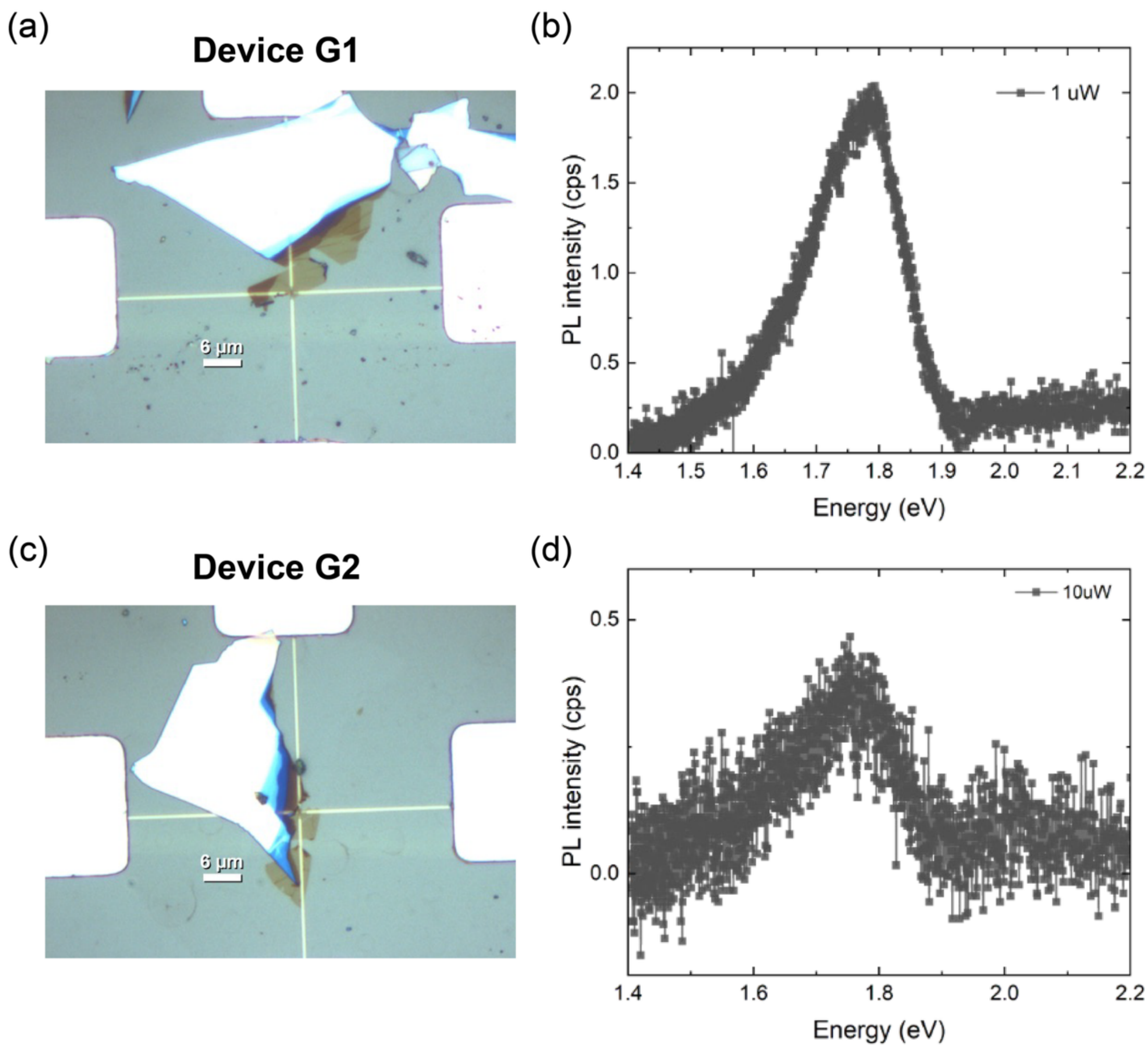

Figure S16. Optical characterization of sub-5 nm nanogap devices fabricated with monolayer MoS_2. (a, c) Optical images of devices G1 and G2. (b, d) Corresponding PL spectra under 532 nm laser irradiation.

To examine the material dependence of nanogap ACEL, identical sub-5 nm nanogap devices were fabricated using monolayer MoS_2 as the active material. The PL spectra of the MoS_2 devices exhibit broad emission centered near 1.77 eV at 3.5 K. In contrast to monolayer WSe_2 devices, no detectable ACEL was observed under the same excitation conditions.

Monolayer WS_2 nanogap control device

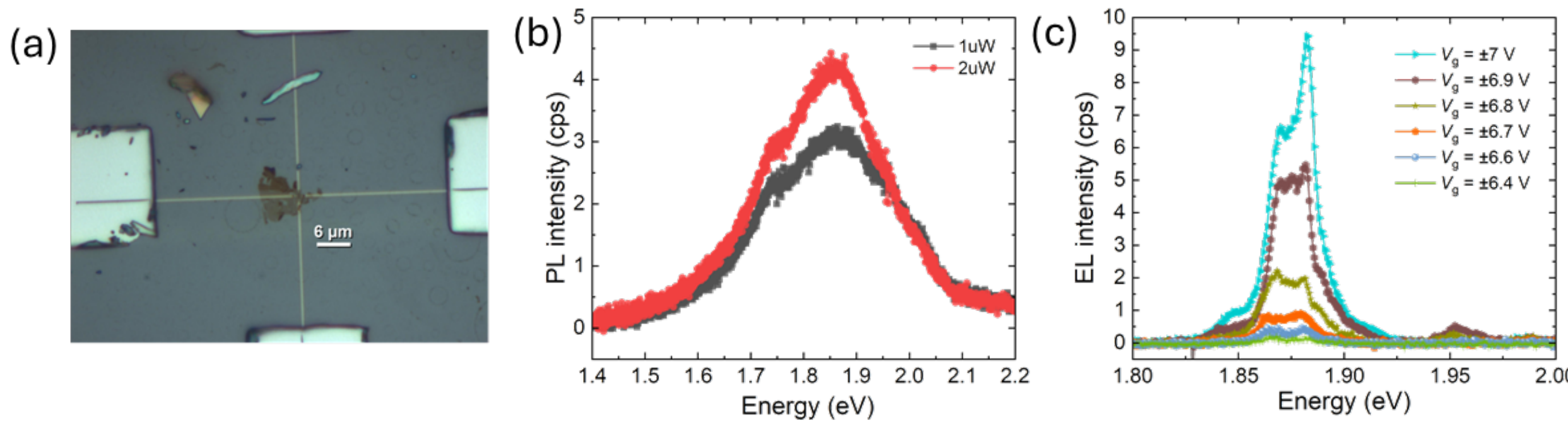

Figure S17. Optical and ACEL characteristics of a monolayer WS_2 sub-5 nm nanogap device. (a) Optical image of the monolayer WS_2 nanogap device. (b) PL spectra centered at ~1.85 eV under 532 nm laser excitation at 1 and 2 μW. (c) Voltage-dependent ACEL spectra measured under direct electrical driving through the dual nanogap electrodes.

Identically structured sub-5 nm nanogap devices were fabricated using monolayer WS_2 as the active material. The PL spectrum exhibits a broad emission band centered at ~1.85 eV. In contrast to the spectrally isolated ACEL observed in WSe_2 devices, the WS_2 device exhibits multiple electrically driven emission features. A dominant ACEL peak appears near 1.88 eV at low driving voltage ($V_g = 6.4$ V), while additional emission features emerge near 1.95 eV as the voltage is increased. These results indicate that carrier injection is not preferentially funneled into a dominant subset of localized states, resulting in spectrally broadened emission behavior compared with WSe_2.

Supporting Information References

(1) Yasutake, Y.; Kono, K.; Kanehara, M.; Teranishi, T.; Buitelaar, M. R.; Smith, C. G.; Majima, Y. Simultaneous fabrication of nanogap gold electrodes by electroless gold plating using a common medical liquid. *Appl. Phys. Lett.* **2007**, *91* (20), 203107.

(2) Serdio V, V. M.; Muraki, T.; Takeshita, S.; Hurtado S, D. E.; Kano, S.; Teranishi, T.; Majima, Y. Gap separation-controlled nanogap electrodes by molecular ruler electroless gold plating. *RSC Adv.* **2015**, *5* (28), 22160–22167.

(3) Choi, Y. Y.; Kwon, A.; Majima, Y. Heteroepitaxial spherical electroless Au-plated Pt-based nanogap electrodes of radius 5 nm and gap separation 0.7 nm. *Appl. Phys. Express* **2019**, *12* (12), 125003.

(4) Serdio V, V. M.; Azuma, Y.; Takeshita, S.; Muraki, T.; Teranishi, T.; Majima, Y. Robust nanogap electrodes by self-terminating electroless gold plating. *Nanoscale* **2012**, *4* (22), 7161–7167.

(5) Lien, D.-H.; Amani, M.; Desai, S. B.; Ahn, G. H.; Han, K.; He, J.-H.; Ager, J. W., III; Wu, M. C.; Javey, A. Large-area and bright pulsed electroluminescence in monolayer semiconductors. *Nat. Commun.* **2018**, *9*, 1229.

(6) Zhao, Y.; Wang, V.; Lien, D.-H.; Javey, A. A generic electroluminescent device for emission from infrared to ultraviolet wavelengths. *Nat. Electron.* **2020**, *3*, 612–621.

(7) Kuner, M. C.; Kaplan, A. D.; Persson, K. A.; Asta, M.; Chrzan, D. C. MP-ALOE: An r2SCAN Dataset for Universal Machine Learning Interatomic Potentials. *arXiv* **2025**, arXiv:2507.05559.

(8) Larsen, A. H.; Mortensen, J. J.; Blomqvist, J.; Castelli, I. E.; Christensen, R.; Dułak, M.; Friis, J.; Groves, M. N.; Hammer, B.; Hargus, C.; et al. The Atomic Simulation Environment—A Python Library for Working with Atoms. *J. Phys.: Condens. Matter* **2017**, *29*, 273002.

(9) Stukowski, A. Visualization and Analysis of Atomistic Simulation Data with OVITO—The Open Visualization Tool. *Modell. Simul. Mater. Sci. Eng.* **2010**, *18*, 015012.

(10) Desai, S. B.; Seol, G.; Kang, J. S.; Fang, H.; Battaglia, C.; Kapadia, R.; Ager, J. W.; Guo, J.; Javey, A. Strain-induced indirect to direct bandgap transition in multilayer WSe_2. *Nano Lett.* **2014**, *14* (8), 4592–4597.
